# The Gb3-enriched CD59/flotillin plasma membrane domain regulates host cell invasion by *Pseudomonas aeruginosa*

**DOI:** 10.1007/s00018-021-03766-1

**Published:** 2021-02-08

**Authors:** Annette Brandel, Sahaja Aigal, Simon Lagies, Manuel Schlimpert, Ana Valeria Meléndez, Maokai Xu, Anika Lehmann, Daniel Hummel, Daniel Fisch, Josef Madl, Thorsten Eierhoff, Bernd Kammerer, Winfried Römer

**Affiliations:** 1 Faculty of Biology, University of Freiburg, Schänzlestraße 1, 79104 Freiburg, Germany; 2 BIOSS, Centre for Biological Signalling Studies, University of Freiburg, Schänzlestraße 18, 79104 Freiburg, Germany; 3 CIBSS, Centre for Integrative Biological Signalling Studies, University of Freiburg, Schänzlestraße 18, 79104 Freiburg, Germany; 4 Center for Biological Systems Analysis, University of Freiburg, Habsburgerstraße 49, 79104 Freiburg, Germany; 5 Spemann Graduate School of Biology and Medicine, University of Freiburg, Albertstraße 19a, 79104 Freiburg, Germany; 6 Department of Biochemistry, University of Geneva, 30 Quai Ernest-Ansermet, 1211 Geneva, Switzerland; 7 Host-Toxoplasma Interaction Laboratory, The Francis Crick Institute, 1 Midland Road, London, NW1 1AT UK; 8 Department of Infectious Disease, MRC Centre for Molecular Bacteriology and Infection, Imperial College London, London, SW7 2AZ UK; 9 Clinic for Vascular and Endovascular Surgery, University Hospital Münster, Albert Schweitzer Campus 1, 48149 Münster, Germany; 10 Present Address: Institute for Experimental Cardiovascular Medicine, University Heart Center Freiburg - Bad Krozingen, and Faculty of Medicine, University of Freiburg, Elsässer Straße 2q, 79110 Freiburg, Germany; 11 Present Address: Institute of Medical Microbiology, Virology and Hygiene, University Medical Center Hamburg-Eppendorf, Martinistraße 52, 20246 Hamburg, Germany

**Keywords:** Host–pathogen interactions, Bacteria, Glycosphingolipid, Lipid rafts, Endocytosis, Signaling

## Abstract

The opportunistic pathogen *Pseudomonas aeruginosa* has gained precedence over the years due to its ability to develop resistance to existing antibiotics, thereby necessitating alternative strategies to understand and combat the bacterium. Our previous work identified the interaction between the bacterial lectin LecA and its host cell glycosphingolipid receptor globotriaosylceramide (Gb3) as a crucial step for the engulfment of *P. aeruginosa* via the lipid zipper mechanism. In this study, we define the LecA-associated host cell membrane domain by pull-down and mass spectrometry analysis. We unraveled a predilection of LecA for binding to saturated, long fatty acyl chain-containing Gb3 species in the extracellular membrane leaflet and an induction of dynamic phosphatidylinositol (3,4,5)-trisphosphate (PIP_3_) clusters at the intracellular leaflet co-localizing with sites of LecA binding. We found flotillins and the GPI-anchored protein CD59 not only to be an integral part of the LecA-interacting membrane domain, but also majorly influencing bacterial invasion as depletion of either of these host cell proteins resulted in about 50% reduced invasiveness of the *P. aeruginosa* strain PAO1. In summary, we report that the LecA-Gb3 interaction at the extracellular leaflet induces the formation of a plasma membrane domain enriched in saturated Gb3 species, CD59, PIP_3_ and flotillin thereby facilitating efficient uptake of PAO1.

**Supplementary Information:**

The online version contains supplementary material available at 10.1007/s00018-021-03766-1.

## Introduction

*Pseudomonas aeruginosa* (PA), a multi-drug resistant bacterium takes a spot on WHO’s highest priority list [1]. This pathogen infects lungs, skin wounds and burns as well as the urinary and gastrointestinal tract of immunocompromised individuals [2, 3]. The very first contact between a pathogenic bacterium and its host cell can already decide the course of infection. Proteins aiding bacteria to successfully colonize epithelial cells are broadly divided into adhesins and invasins. The two lectins of PA, namely, LecA and LecB, were initially defined as exemplary adhesion proteins [4], however, several reports now suggest an additional role of LecA in the pathogenicity of PA [5–8]. The homotetrameric protein is localized to the outer membrane of the bacterium [9] and preferentially binds to the glycosphingolipid (GSL) globotriaosylceramide (Gb3; also known as CD77 or the P^k^ blood group antigen). Gb3 is the receptor for the B-subunit of AB_5_ toxins produced by *Shigella dysenteriae* and enterohemorrhagic strains of *Escherichia coli* (Shiga toxin and Shiga-like toxin B-subunit, respectively, here referred to as StxB) [10]. We previously demonstrated the importance of the interaction between Gb3 and LecA for PA uptake into lung epithelial cells [8]. Moreover, a novel divalent LecA ligand identified from a galactoside-conjugate array, bound to the lectin with high affinity and lowered the invasiveness of PA by up to 90% [11].

Pathogens hijack GSLs to induce plasma membrane bending and to transduce signals in order to promote endocytosis [12, 13]. Both PAO1 and its lectin LecA trigger the activation of CrkII, an adaptor protein implicated in various cellular processes including cell adhesion and cytoskeletal reorganization [14, 15], by phosphorylation at Tyr^221^ [16, 17]. Interestingly, induction of this signaling pathway is dependent on the binding of LecA to Gb3 located in the extracellular leaflet of the plasma membrane. Clearly, these observations raise the question of how the signal generated by receptor binding at the extracellular membrane leaflet, is transmitted to the intracellular site, activating proteins such as CrkII. One possibility could be the involvement of GSLs with long fatty acyl chains interdigitating into the inner leaflet of the membrane bilayer [18–20]. Additionally, the local lipid and protein environment of Gb3 may affect the induced signaling events and endocytic trafficking routes. The lipid environment as well as length and saturation level of the fatty acyl chains of GSLs influence orientation and accessibility of the carbohydrate groups and affect the binding behavior of StxB to Gb3 in model membrane systems [21–23]. Ordered domains in the outer leaflet of the cellular plasma membrane are often termed lipid rafts. These domains are enriched in GSLs, cholesterol and glycosylphosphatidylinositol (GPI)-anchored proteins and serve as sorting and signaling platforms [24–26]. Due to the high saturation level of the fatty acyl chains of Gb3, the lipid is preferentially but not exclusively located in lipid rafts [23, 27, 28].

Many bacterial species evade the immune system by invading their target host cell. The pathogen's entry mechanisms comprise an astonishing variety of possibilities. One of the most common modalities of pathogen entry into the host cell is by exploiting specialized plasma membrane domains [29, 30]. Flotillin membrane domains consist of heterotetrameric protein complexes of flotillin-1 and flotillin-2 [31, 32], that anchor to the cytosolic face of the plasma membrane and to endosomal structures via myristoylation and palmitoylation [33]. Flotillins have been implicated in cell adhesion, vesicle trafficking as well as cytoskeleton rearrangement and are commonly known to scaffold lipid rafts [34–37]. Importantly, flotillin-enriched domains also represent active cellular signaling platforms typically involving Src family kinases [38, 39]. A role of flotillins in endocytosis was first described for the GPI-anchored complement inhibitor protein CD59 and the GSL GM1, the cholera toxin receptor [40], and later also for the amyloid precursor protein, glutamate- and dopamine-transporters [41, 42]. In addition, flotillins have been implicated in bacterial uptake processes [43, 44].

The search for novel therapeutic approaches for multi-drug-resistant bacteria is driven by the complexity of bacterial infections, host–pathogen interaction dynamics and the rapid adaptation capacity of bacteria. In this study, we focus on the virulence factor LecA to better understand initial processes of host–pathogen interactions. So far, little is known about important players within and associated to the Gb3-enriched plasma membrane domain leading to the induction of signaling and endocytosis of PA via its lectin LecA. We characterize both proteins and lipids of this membrane domain and identify interaction partners of LecA and Gb3 using a pull-down strategy followed by mass spectrometry (MS) analysis. We unravel an involvement of CD59, flotillins, Src family kinases and phosphatidylinositol (3,4,5)-trisphosphate (PIP_3_) in the process of LecA binding to the plasma membrane. As major finding, we demonstrate that CD59 and flotillins promote PA invasion into lung epithelial cells. Deepening our understanding of the interplay between virulence factors and the host cell plasma membrane is crucial to develop novel treatment strategies and to minimize the rising risk of untreatable bacterial infections.

## Materials and methods

### Cell culture and lectin stimulation

The human lung epithelial cell line H1299 (American Type Culture Collection, CRL-5803) was cultured in Roswell Park Memorial Institute (RPMI) medium supplemented with 10% fetal calf serum (FCS) and 2 mM l-glutamine at 37 °C and 5% CO_2_. LecA was expressed in *E. coli* BL21 (DE3), transformed with the plasmid pET25-pa1l encoding the lectin and purified as published in Ref [7]. The B-subunit of Shiga toxin 1 (StxB) was bought from Sigma Aldrich. Lyophilized StxB was resuspended in ultrapure water, while LecA was diluted in calcium- and magnesium-free Dulbecco’s phosphate-buffered saline (DPBS−/−). Both lectins were filtered sterile and stored at 4 °C. Cells were stimulated with a final concentration of 100 nM LecA or StxB for indicated time points. For cholesterol depletion, cells were pre-treated with 10 mM methyl-beta-cyclodextrin (MβCD) in RPMI for 30 min at 37 °C and washed once with DPBS−/− before lectin stimulation.

### SDS-PAGE and immunoblot analysis

Stimulated H1299 cells were harvested in radio-immunoprecipitation assay (RIPA) buffer [20 mM Tris (pH 8.0), 0.5% (w/vol) sodiumdeoxycholate, 13.7 mM NaCl, 10% (vol/vol) Glycerol, 0.1% (w/vol) SDS, 2 mM EDTA, freshly supplemented with phosphatase and protease inhibitors (Sigma)] for 60 min at 4 °C. Protein concentrations were determined by Pierce BCA Protein Assay Kit (Thermo Fisher Scientific). Cell lysates were denatured in SDS loading buffer and boiled at 95 °C for 7 min. Equal amounts of proteins were separated on 8 or 12% Tris–glycine gels and transferred on a nitrocellulose membrane by application of 0.12 A/gel. BSA (3% in TBS-T [50 mM Tris (pH 7.6), 154 mM NaCl, 0.5% (vol/vol) Tween 20)] was used to inhibit unspecific binding to the membrane before primary antibodies (1:1000) were added at 4 °C overnight. The corresponding horseradish peroxidase-conjugated secondary antibody (1:2000) was incubated with the membrane for another hour at room temperature (RT) the following day. Finally, Clarity^TM^ Western ECL Chemiluminescent Substrate (Bio-Rad) was used to detect the luminescence signal by a Vilber Lourmat Fusion FX chemiluminescence imager. For the detection of LecA-biotin, an IRDye 800CW Streptavidin antibody (926–32230) was used and fluorescence signal was measured by the Odyssey CLx (LI-COR). Protein levels were analyzed by means of Fiji ImageJ 1.0 software and gel analysis tools.

### Pull-down and immunoprecipitation

For pull-down studies, lectins were biotinylated by NHS-ester conjugation (Thermo Fisher Scientific) and dialyzed against DPBS-/- overnight. H1299 cells treated with 100 nM LecA-biotin or StxB-biotin for indicated time points were lysed by a buffer composition consisting of 25 mM Tris/HCl (pH 7.4), 150 mM NaCl, 1 mM EDTA, 1% NP40 and 5% glycerol for 60 min at 4 °C. After a short clarification centrifugation step, normalized protein lysates were incubated with magnetic streptavidin beads (Thermo Fisher Scientific) for 3 h at 4 °C. Subsequently, beads were washed three times with lysis buffer and in between rotated for 10 min at 4 °C. Pulled-down proteins were eluted with 2 × SDS loading buffer and subjected to immunoblot analysis for further characterization. For co-immunoprecipitation, the Capturem Protein A technology from Takara was employed according to the manufacturer’s protocol. Briefly, H1299 cells were stimulated with LecA as described above and lysed by the provided lysis buffer freshly supplemented with protease inhibitor cocktail. Cell lysates were incubated with the target primary antibody for 60 min at 4 °C and end-to-end rotation. The antibody/antigen complex was loaded on equilibrated spin columns, washed and eluted in elution buffer. Finally,  SDS loading buffer was added to the eluate and samples were boiled and analyzed by immunoblot analysis as described. For depletion of GSLs, cells were cultivated for 4 days in the presence of 2.5 μM d-threo-l-phenyl-2-palmitoylamino-3-morpholino-l-propanol (PPMP; Santa Cruz) to inhibit synthesis of glucosylceramide-based GSLs [45].

### Lipid analysis by MS

Untreated or MβCD-treated H1299 cells were stimulated with biotinylated lectins for 20 min at 37 °C. Pull-down was performed as described above with the exception that streptavidin beads were finally eluted in methanol/water (1:1) and stored at − 80 °C until further usage. Lipid extraction by centrifugation with chloroform (containing 1.5 ng/µl heptadecanoic acid as internal standard) was performed as previously described [46]. Lipids were reconstituted in isopropanol:acetonitrile:water (2:1:1). Initial experiments were assessed by high-resolution mass spectrometry (Synapt G2Si, Waters Corporation) operated in positive MS^E^ mode. Displayed data including the Gb3 profile of H1299 cells and Gb3 species of pull-down experiments were measured by targeted LC-QqQ-MS (6460, Agilent Technologies) operated in SRM/MRM mode. Chromatographic separation was achieved on a BEH C18 column (100 mm × 2.1 mm, 1.8 µm, Waters Corporation) as previously described [47]. For evaluation of the lectin preferences, cell lysates of unstimulated, LecA- and StxB-stimulated H1299 cells were split into two fractions: 10% were used to assess the Gb3 levels present in the individual sample (input). The remaining 90% of the lysates were used for pull-down experiments. Obtained values were normalized to the internal standard, to their corresponding input values and, additionally, to correct for different binding strengths, to the species Gb3(d18:1/16:0).

### Protein analysis by MS

The pull-down of H1299 cells stimulated with LecA for 5 and 15 min was conducted as described above. After incubation of the cell lysates with magnetic streptavidin beads, beads were washed three times with lysis buffer. Further processing for protein MS was performed at Toplab GmbH in Planegg-Martinsried. The samples were submerged in cleavage buffer (8 M urea/0.4 M NH_4_HCO_3_ buffer) and reduced with 5 μl of 45 mM dithiothreitol for 30 min at 55 °C before they were alkylated with 5 μl of 100 mM iodoacetamide for 15 min at RT in the dark. For trypsin digestion, the samples were diluted to 2 M urea/0.1 M NH_4_HCO_3_ with 140 μl of HPLC grade water (VWR). Digestion with mass-spec grade trypsin (Serva, porcine) was performed at 37 °C overnight. For nano-LC–ESI–MS/MS, the digests were acidified to 0.05% FA and 10 μl of the digests were subsequently injected. HPLC separation was done using an EASY-nLC1000 (Thermo Scientific) system with the following columns and chromatographic settings: the peptides were applied to a C18 column (Acclaim PepMap 100 pre-column, C18, 3 μm, 2 cm × 75 μm Nanoviper, Thermo Scientific) and subsequently separated using an analytical column (EASY-Spray column, 50 cm × 75 μm ID, PepMap C18 2 μm particles, 100 Å pore size, Thermo Scientific) by applying a linear gradient (A: 0.1% formic acid in water, B: 0.1% formic acid in 100% ACN) at a flow rate of 200 nl/min. The gradient used was: 1–25% B in 120 min, 25–50% B in 10 min, 84% B in 10 min.

MS analysis was conducted on a LTQ Orbitrap XL mass-spectrometer (Thermo Scientific), which was coupled to the HPLC-system. The mass spectrometer was operated in the so-called “data-dependent” mode where after each global scan the five most intense peptide signals are chosen automatically for MS/MS-analysis.

The LC–ESI–MS/MS data were used for a database search with the software Mascot (Matrix Science) using the SwissProt database, species: human. Peptide mass tolerance was set to 50 ppm, fragment mass tolerance was set to 0.6 Da, a significance threshold *p* < 0.05 was used. Carbamidomethylation at cystein was set as fixed modification; oxidation at methionine was set as variable modification. Peptides with up to 1 missed cleavage site were searched. Identified proteins are sorted by the Exponentially Modified Protein Abundance Index (emPAI), which offers approximate, label-free, relative quantitation of the proteins in a mixture based on protein coverage by the peptide matches in a database search result [48].

### Lectin labeling and immunofluorescence microscopy

Alexa Fluor 488 or Alexa Fluor 647 dyes (Thermo Fisher Scientific) were used to label the lectins according to the manufacturer’s protocol. Hereby, the ratio of dye to lectin was 5:1. H1299 cells were seeded on glass coverslips and allowed to adhere. The next day, cells were stimulated with fluorescently labeled LecA for indicated time points. For the inhibition of phosphoinositide 3-kinase (PI3)-kinases, cells were treated with 100 nM Wortmannin (Sigma-Aldrich) 30 min prior and during stimulation with LecA. Subsequently, cells were fixed with 4% formaldehyde for 15 min at RT and/or subjected to ice-cold methanol for 8 min at − 20 °C. The membrane was permeabilized by 0.2% TritonX-100 in DPBS-/-, if the cells were not treated with methanol. Fixed cells were blocked in 3% BSA in DPBS-/- for 30 min and incubated with target primary antibodies (1:100) for 1 h at RT. After three washes, cells were stained with fluorescently labeled secondary antibodies (1:200) for 30 min at RT in the dark. Nuclei were counterstained with DAPI (1:1000) and samples were mounted on cover slips using Mowiol (containing the anti-bleaching reagent DABCO). Samples were imaged by means of a confocal laser scanning microscope system from Nikon (Eclipse Ti-E, A1R), equipped with a 60 × oil immersion objective and a numerical aperture of 1.49. Co-localization was calculated in Fiji ImageJ 2.0.0 software using the Coloc2 plugin. A minimum of three biological replicates with ≥20 cells per condition were analyzed.

### G-LISA

Rac1 activation was measured by means of a G-LISA Activation Assay with luminescence read-out (Cytoskeleton, Inc.) according to the manufacturer’s protocol. Briefly, LecA-treated and snap frozen cell lysates were subjected to a Rac-GTP affinity 96-well plate and incubated for 30 min. Following a 2 min incubation with antigen presenting buffer, anti-Rac1 antibody was added and the plate was vigorously shaken for 45 min at RT. The procedure was repeated with a corresponding secondary antibody. Subsequently, the luminescence signal was detected using a HRP detection reagent and a microplate reader (Synergy H4, Biotek).

### Plasmid transfection

H1299 cells were transfected with 1 μg plasmid for single transfections and 0.5 μg for co-transfection of two plasmids. Lipofectamine 2000 was used as transfection reagent (Thermo Fisher Scientific). Cells were kept in Opti-MEM Reduced Serum Media (Thermo Fisher Scientific) during the 3 h incubation before medium was exchanged to RPMI. The plasmid pcDNA3-AKT-PH-GFP [Addgene #18,836 (Craig Montell)] was requested from the Signaling Factory of the Albert-Ludwigs-University Freiburg. Flotillin-1- and flotillin-2-mCherry were kind gifts from A. Echard (Institut Pasteur, Paris, France).

### Live-cell imaging

H1299 cells were grown on glass cover slips (Thermo Fisher Scientific) until 80% confluency. On the day of the experiment, cells were pre-washed with and kept in Hanks’ Balanced Salt Solution (Thermo Fisher Scientific) while imaging. Live cell imaging was performed at 37 °C using an incubator stage (Okolab) mounted onto a confocal laser scanning microscope (Nikon Eclipse Ti-E, A1R).

### Creation of Δ*FLOT1* cell lines with CRISPR/Cas9 system

Knockout of *Flotillin-1* was accomplished according to the protocol previously described [49]. Briefly, single guide RNAs (sgRNAs, designed using crispr.mit.edu, listed in Table S1) were cloned into pX458 vector (Addgene plasmid #48138) and transfected into H1299 cells. After 72 h, the transfected cells were trypsinized and resuspended in FACS buffer. GFP positive cells were sorted into 96-well plates. After 2 weeks, single cell clones were expanded into bigger culture dishes. The initial screening for knockout clones was conducted by immunoblotting against flotillin-1 antibody. The genomic locus was amplified by PCR and analyzed by Sanger sequencing to confirm the knockout.

### Quantitative RT-PCR

The single clones were subjected to TRIzol (Sigma-Aldrich) treatment to extract mRNA. Subsequently, the extracted mRNA was transcribed into cDNA using the Maxima First Strand cDNA Synthesis kit. SYBR Select Master Mix for CFX was used for qPCR according to the manufacturer’s protocol. Samples were analyzed using the CFX384 qPCR system (Bio-Rad, version 3.0). Relative flotillin mRNA levels were normalized to GAPDH (glyceraldehyde 3-phosphate dehydrogenase) mRNA levels. Primer sequences are listed in Table S1.

### siRNA transfection

H1299 cells were depleted by means of on-target SMART pools of siRNA against flotillin-2 (Cat. No. L-003666-01-0010) or CD59 (L-004537-02-0005), purchased from Dharmacon, Horizon Discovery. The siRNA against caveolin-1 was from Santa Cruz (Cat. No. sc-29241). During the incubation with a mixture of siRNA and Lipofectamine 2000, cells were kept in Opti-MEM Reduced Serum Media. Sequences of the siRNA are listed in Table S1.

### Invasion assay

*P. aeruginosa* strain PAO1, characterized first in 1955 [50], was cultivated, GFP-tagged and deleted of LecA as described before [8]. For the invasion assay, overnight cultures of PAO1 wild-type (WT) and ΔLecA were centrifuged before resuspension of the pellet in RPMI containing 1 mM CaCl_2_ and MgCl_2_. H1299 cells (with control siRNA (Qiagen), CD59- or flotillin-depleted) were treated with PA at a multiplicity of infection (MOI) of 100 for 2 h at 37 °C. After washing with PBS, cells were treated with Amikacin sulphate (400 μg/ml; Sigma-Aldrich) for 2 h at 37 °C to exclude extracellular bacteria. Finally, the cells were lysed with 0.25% (vol/vol) Triton X-100, plated on LB-Agar plates containing 60 μg/ml Gentamicin and incubated at 37 °C overnight. The following day, colonies were counted and invasion rate was calculated as percentage of Amikacin-survived bacteria to the total number of bacteria not treated with Amikacin. Mean values of three individual experiments were normalized to the invasion rate of WT PAO1 into WT H1299.

### Antibodies and chemical reagents

The following antibodies were obtained from commercial sources: monoclonal mouse anti-CD59 (MEM-43, Abcam, Cat. No. ab9182), monoclonal rabbit anti-flotillin-1 (D2V7J) XP (Cell Signaling, Cat. No. 18634), monoclonal mouse anti-flotillin-2 (BD Biosciences, Cat. No. 610383), polyclonal rabbit anti-GAPDH (Sigma, Cat. No. G9545), monoclonal rabbit anti-phospho-Src Family (Tyr^416^, Cell Signaling, Cat. No. 6943), monoclonal rabbit anti-Src Family (Cell Signaling, Cat. No. 2109), monoclonal rabbit anti-caveolin-1 **(**D46G3**)** XP (Cell Signaling, Cat. No. 3267). LecA was detected by a custom-made polyclonal rabbit anti-LecA antibody (Eurogentec, France).

The protease inhibitors Aprotinin, Leupeptin, Pefablock, Sodium orthovanadate and Phosphatase Inhibitor Cocktail 3 were all obtained from Sigma-Aldrich. RPMI 1640, DPBS-/-, FCS and l-Glutamine were all purchased from Gibco (Thermo Fisher Scientific). The following chemicals were obtained from Roth: BSA, DABCO, DAPI, EDTA, glycerol, LB, Mowiol, NaCl, sodium deoxycholate, NH_4_Cl, paraformaldehyde, SDS, Tris (hydroxymethyl)-aminoethane, Triton X-100 and Tween 20. Amikacin sulphate and Gentamicin were obtained from Sigma-Aldrich. StxB was purchased from Sigma-Aldrich (Cat. No. SML0562).

### Statistical analysis

All data in graphs are presented as mean ± standard deviation (SD) and were calculated from the results of independent experiments. Statistical testing was performed with GraphPad Prism software using data of ≥ 3 biological replicates. When appropriate, one-way analysis of variance (ANOVA), two-way ANOVA or two-tailed unpaired t-test was conducted to determine the significance of the data. Tests with a *p *value < 0.05 are considered statistically significant and marked by asterisks.

## Results

### LecA preferentially binds to Gb3 species with saturated fatty acyl chains

**Fig. 1:**
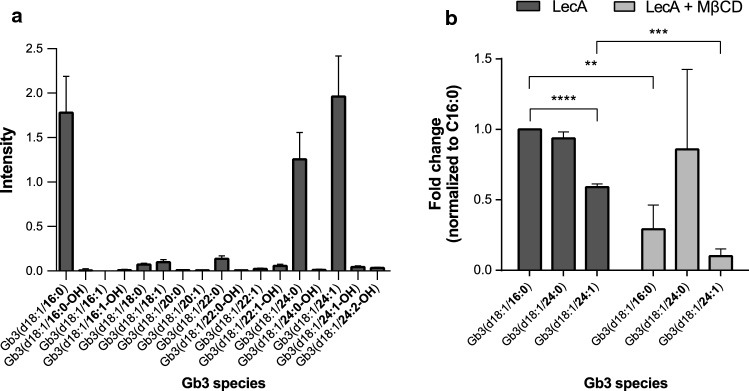
Lipid analysis by LC–MS reveals a preference of LecA for saturated Gb3 species. **a** Distribution of Gb3 species in H1299 cells. Most dominantly present Gb3 species were Gb3(d18:1/16:0), Gb3(d18:1/24:0) and Gb3(d18:1/24:1). Intensities were normalized to an internal standard. **b** Pulled-down Gb3 species [normalized to internal standard, input values and Gb3(d18:1/16:0)] by LecA-biotin demonstrated a preference for saturated over unsaturated Gb3 species. Significantly less Gb3(d18:1/16:0) and Gb3(d18:1/24:1) was pulled-down in MβCD-treated cells stimulated with LecA-biotin. The Gb3 species Gb3(d18:1/24:0) seemed less affected by the treatment. Still, the unsaturated species Gb3(d18:1/24:1) was least preferred. For clarity, only the three dominantly present species Gb3(d18:1/16:0), Gb3(d18:1/24:0) and Gb3(d18:1/24:1) are depicted. For all panels: bars display mean values of three biological replicates, error bars represent SD, **p < 0.01, ***p < 0.001, ****p < 0.0001 (one-way ANOVA and Dunnett’s multiple comparisons test)

The length and saturation level of Gb3 affects binding and subsequent trafficking of StxB [22, 51], however, the influence on LecA binding is not known. First, we characterized the Gb3 species dominantly present in our cell model, the lung epithelial cell line H1299. This cell model was chosen due to the well-known impact of PA in chronic lung infections [52]. The cells were lysed in a 1:1 mixture of methanol and water. Lipids were isolated by chloroform purification and characterized by MS. Approximately 36% of the Gb3 species present were assigned to the unsaturated species Gb3(d18:1/24:1). Around 32% were matched to the saturated species Gb3(d18:1/16:0) and 23% to Gb3(d18:1/24:0) (Fig. 1a). Additionally, we could detect small traces of Gb3(d18:1/18:0), Gb3(d18:1/18:1), Gb3(d18:1/22:0) and Gb3(d18:1/22:1) and hydroxylated versions of the mentioned species (9% together). To better understand the influence of Gb3 length and saturation degree on the binding of LecA, we developed a pull-down strategy with subsequent targeted liquid chromatography (LC)–MS analysis (Fig. S1). We used biotinylated LecA or StxB to stimulate H1299 cells for 20 min. Lectin-bound membrane fragments were isolated using streptavidin beads before Gb3 species pulled-down together with biotinylated LecA or StxB were assessed by MS. In comparison to the tetrameric LecA, higher numbers of all detected Gb3 species were measured for the pentameric StxB (Fig. S2b), which can be explained by its binding capacity of up to 15 Gb3 lipids while LecA exhibits only four binding sites. The normalization to Gb3(d18:1/16:0) species allowed a comparison independent of binding avidity and demonstrated very similar binding preferences for both lectins (Fig. 1b for LecA and S2a for StxB). Interestingly, a clear prevalence for Gb3 species with saturated fatty acyl chains [Gb3(d18:1/16:0) and Gb3(d18:1/24:0)] in comparison to the unsaturated Gb3(d18:1/24:1) was unraveled for both lectins, while no significant difference between Gb3(d18:1/16:0) and Gb3(d18:1/24:0) was detected. Importantly, the same trend was observed for the less represented Gb3 species (Figs. S2c and S2d).

Lipid rafts are known to comprise of GSLs and enriched levels of cholesterol [27]. We studied the impact of cholesterol on the binding behavior of LecA to Gb3 by depleting H1299 cells of cholesterol by MβCD treatment (10 mM, 30 min). The treatment led to a significant cholesterol reduction in the input samples (Fig. S2e). Subsequently, we addressed the question whether cholesterol depletion alters the Gb3 binding preference of LecA. Of note, the treatment significantly diminished overall binding to Gb3 (Fig. 1b). The strongest effect was observed for the species Gb3(d18:1/16:0) and Gb3(d18:1/24:1), whose binding was significantly reduced as compared to untreated cells. However, the same cannot be claimed for the saturated species Gb3(d18:1/24:0) as it displayed high variability between experiments. Interestingly, the unsaturated species Gb3(d18:1/24:1) remained least accessible for LecA. Similar results were obtained for StxB (Fig. S2a).

To assess any unspecific binding of lipids to the beads, unstimulated H1299 cells were used as control in each condition. A heat-map displaying all significantly changed lipids is depicted in Fig. S3 and demonstrates the clear increase in pulled-down lipids for the lectin-treated conditions as compared to the unstimulated control. Our experiments, therefore, revealed a preference of the two lectins, LecA and StxB, for saturated over unsaturated Gb3 species. Overall binding to Gb3, but not the preference for saturated Gb3 species was influenced by cholesterol depletion.

### Lipid raft proteins define the binding domain of LecA at the plasma membrane

The importance of lipids in endocytosis is not yet fully understood. However, various proteins are integral and inevitable players of the uptake process. These proteins can directly interact with the ligand to establish attachment points, trigger signaling pathways, scaffold the binding domain, or aid in membrane bending and scission processes. We aimed at identifying novel protein interaction partners of LecA within the Gb3 membrane domain following stimulation of H1299 cells with LecA-biotin for 5 and 15 min. The lysed membrane fragments were incubated with streptavidin beads before pulled-down proteins were on-bead trypsin-digested and analyzed by MS. The protein hits included various cytoskeletal components and cytoskeleton-regulating proteins (Table S2). Already after 5 min of LecA incubation, vimentin was highly enriched in the LecA-treated sample and after 15 min of stimulation, actin, tubulin, myosin-9 and the small GTPase Rac1 were detected. Additionally, the GPI-anchored protein CD59 and the Src kinase Yes were identified. Together with the scaffolding protein flotillin-1, which accumulated after 15 min, these proteins represent well-reported lipid raft components [53].

**Fig. 2:**
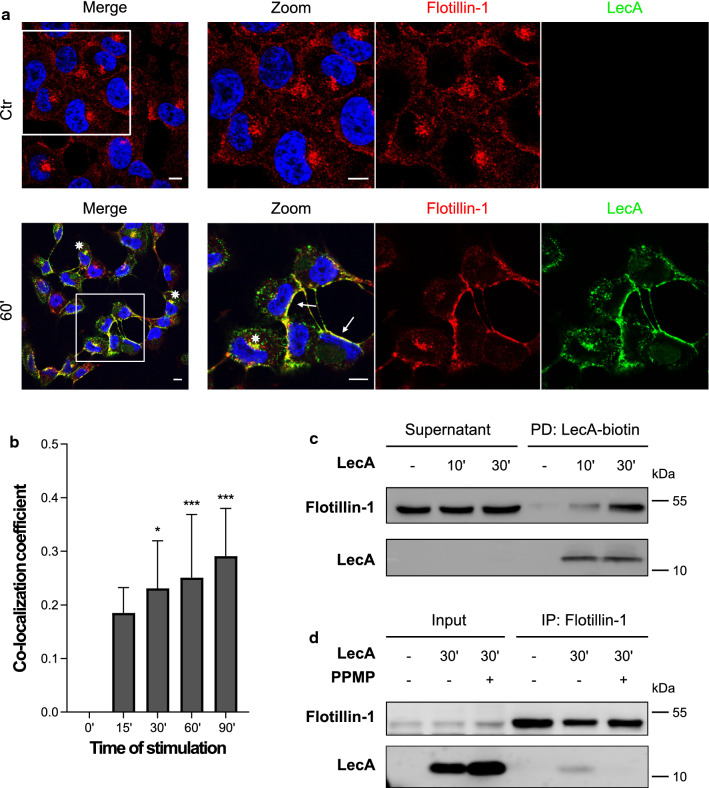
Flotillins are recruited to the plasma membrane upon LecA stimulation. **a** Fluorescence co-localization studies of flotillin-1 (red) and LecA (green) after 60 min of lectin stimulation. Nuclei were counterstained by DAPI. Framed areas were magnified. White arrows point at co-localization events at the plasma membrane, asterisks at perinuclear co-localization. Scale bar: 10 μm. **b** Mander’s co-localization coefficient quantified between the fluorescence signals of flotillin-1 and LecA were statistically compared to time point 0. Bars display mean values of at least three biological replicates, error bars represent SD, *p < 0.05, ***p < 0.001 (one-way ANOVA and Dunnett’s multiple comparisons test). **c** Pull-down of LecA-biotin resulted in a time-dependent enrichment of flotillin-1. **d** LecA was co-immunoprecipitated with flotillin-1 after 30 min of LecA treatment, the binding and precipitation was inhibited by PPMP treatment

**Fig. 3:**
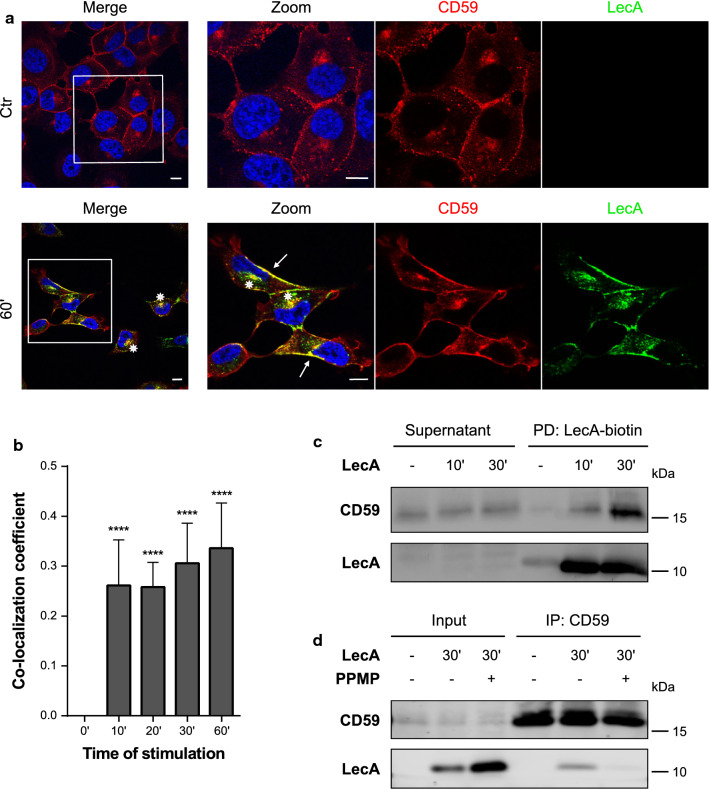
The LecA plasma membrane domain includes CD59 proteins. **a** Fluorescence co-localization studies of CD59 (red) and LecA (green) after 60 min of lectin stimulation. Nuclei were counterstained by DAPI. Framed areas were magnified. White arrows point at co-localization events at the plasma membrane, asterisks at perinuclear co-localization. Scale bar: 10 μm. **b** Signal overlay of LecA and CD59 is displayed by Mander’s co-localization coefficient and statistically compared to time point 0. Bars display mean values of at least four biological replicates, error bars represent SD, ****p < 0.0001 (one-way ANOVA and Dunnett’s multiple comparison tests). **c** CD59 was validated as component of the LecA-binding domain by pull-down of LecA-biotin. **d** Immunoprecipitation of CD59 co-precipitated LecA after 30 min of stimulation. PPMP treatment to deplete cells in glucosylceramide-based GSLs, such as Gb3, largely inhibited the precipitation of LecA

To characterize the identified hits in more detail, we performed confocal microscopy, co-immunoprecipitation (co-IP) and immunoblotting experiments (Figs. 2 and 3). Fluorescently labeled LecA co-localized with flotillin-1 (Fig. 2a), flotillin-2 (Fig. S4a), and CD59 (Fig. 3a) in immunofluorescence studies. The fluorescence signal overlapped predominantly at the plasma membrane (see white arrows in Figs. 2a, 3a and S4a) but also in vesicular structures and perinuclear regions (marked by asterisks in Figs. 2a, 3a and S4a). In contrast to CD59, flotillins were detected almost exclusively in the perinuclear region in untreated control cells but re-localized to the plasma membrane upon LecA treatment (Figs. 2a and S4a). Co-localization was quantified and revealed an increasing overlap between LecA and flotillins or CD59 over time (Figs. 2b, 3b and S4b).

Additionally, we performed pull-down studies of LecA-biotin and confirmed the presence of the two flotillins and CD59 in the LecA plasma membrane domain biochemically by immunoblotting (Figs. 2c, 3c and S4c). Of note, the pull-downs suggest a time-dependent recruitment of the proteins to the binding site indicated by increasing protein levels over time. Both, flotillin-1 and CD59 could be detected together with LecA in the eluates of the corresponding co-IP of LecA-treated H1299 cell lysates (Figs. 2d and 3d). The co-precipitation was glycan-dependent since PPMP, an agent that inhibits the synthesis of glucosylceramide-based GSLs [45], strongly diminished the interaction of LecA with flotillin-1 or CD59. However, we did not witness a direct binding of LecA to glycans on flotillin-1 or CD59 as shown by immunoblots of IP eluates, which were incubated with LecA-biotin and fluorescent streptavidin (Fig. S5). We, therefore, conclude that LecA binding to Gb3 induces the recruitment of flotillins and CD59 into LecA/Gb3 membrane domains.

We also tested for interactions between the lipid raft marker caveolin-1 and LecA (Fig. S6). The two proteins partiallyco-localized at the plasma membrane, however, quantified values for co-localization of LecA with caveolin-1 were 38% lower than the calculated values for LecA with flotillin-1 at 60 and 90 min of stimulation (Figs. 2b and S6b). The quantified co-localization between LecA and CD59 at 60 min was more than 50% higher compared to co-localization of LecA with caveolin-1 (Fig. 3b). In caveolin-1-depleted H1299 cells, co-localization between LecA and flotillin-1 was significantly reduced (Fig. S7). In addition, the observed re-localization of flotillins to the plasma membrane upon LecA stimulation was attenuated in the caveolin-1 depletion condition. Compared to WT H1299 cells, less binding and uptake of LecA into caveolin-1-depleted cells was observed (Fig. S7c).

### LecA activates Src kinases and Rac-1

**Fig. 4:**
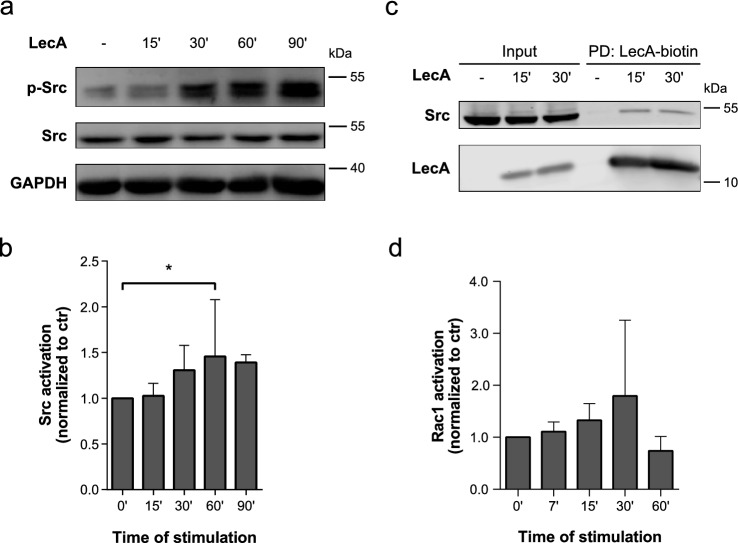
Src family kinases and Rac1 are activated upon LecA stimulation. **a** Src family kinases are phosphorylated upon LecA treatment of H1299 cells as demonstrated by immunoblot analysis. **b** Src activation was quantified by the ratio between phospho-Src (Tyr^416^) and Src and normalized to control levels. Bars display mean values of at least four biological replicates, error bars represent SD, *p < 0.05 (one-way ANOVA and Dunnett’s multiple comparisons test). **c** The Src family kinase was pulled-down together with LecA after 15 and 30 min of treatment. **d** G-LISA of Rac1 demonstrated a non-significant activation of the small G protein Rac1 upon LecA stimulation. Values were normalized to control levels. Bars display mean values of three biological replicates, error bars represent SD

We recently demonstrated that the adaptor protein CrkII is activated upon stimulation with LecA in H1299 cells [17]. Src family kinases were identified as a crucial upstream factor of LecA-induced CrkII activation. Since the Src kinase Yes was one of the hits detected in the protein MS analysis, we further analyzed the role of Src family kinases in processes related to LecA binding. Using immunoblot analysis, we confirmed the LecA-mediated increase in phosphorylation of Src kinases at Tyr^416^ in a time-dependent manner (Fig. 4a, b, also compare with [17]). We also validated the presence of Src in the LecA plasma membrane domain by pull-down of LecA-biotin (Fig. 4c). We performed Rac1 G-LISA to analyze the role of Rac1, a multifunctional GTPase found in our MS screen (Fig. 4d). The small GTPase was activated upon LecA stimulation, however, great variability between the biological replicates was observed. Potentially, this was owed to the short lifetime of Rac1 activation.

Taken together, our data demonstrate that LecA assembles a flotillin/CD59-enriched plasma membrane domain and induces the recruitment of Src kinases and the small GTPase Rac1 for the initiation of cellular mechanisms. These factors in combination with the PI3-kinase signaling cascade represent critical players in the process of actin reorganization [54–56].

### PIP_3_ clusters are induced upon LecA treatment

**Fig. 5:**
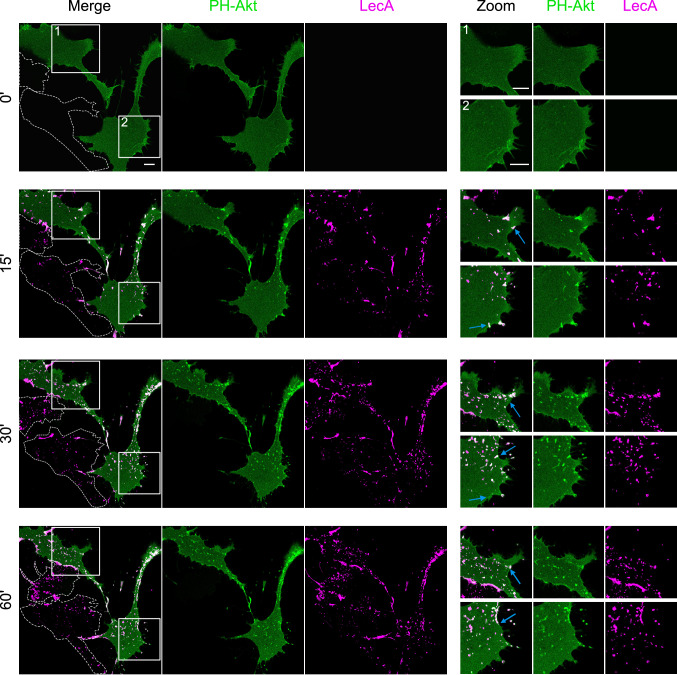
LecA induces clustering of PIP_3_ in H1299 cells. **a** Left panel: time-lapse images of PH-Akt-GFP expressing H1299 cells exposed to fluorescent LecA over 60 min. On the left side, LecA binds two untransfected cells (traced in grey). Right panel: magnifications of framed areas highlight dynamic co-localizing events between LecA and PH-Akt-GFP correlating with LecA endocytosis (marked by blue arrows). Scale bar: 10 μm

Phosphatidylinositols (PIPs) naturally bear a long saturated C18 acyl chain and, therefore, can reach across the bilayer enabling an interaction with long chains of lipids in the extracellular leaflet [57, 58]. To investigate a potential coupling mechanism between the extracellular and the intracellular leaflet of the host cell plasma membrane, we studied the influence of LecA-treatment on phosphatidylinositol (4,5)-bisphosphate (PIP_2_) phosphorylation in H1299 cells. The PH domain of the PI3-kinase downstream signaling molecule Protein kinase B (Akt) can sense PIP_3_ and is commonly used as a GFP-fusion protein to study PIP_3_ dynamics in living cells [59, 60]. We transfected H1299 cells with PH-Akt-GFP and traced GFP signal for 60 min following fluorescent LecA-treatment. Prior to stimulation with LecA, cells displayed an evenly distributed GFP signal in the cytosol and at the plasma membrane (Fig. 5, upper panel). Upon stimulation with LecA, PH-Akt-GFP clusters appeared at LecA binding sites indicating the phosphorylation of PIP_2_ through activated PI3-kinases and clustering of PIP_3_ in the intracellular leaflet of the plasma membrane (Fig. 5, lower panels). The first PIP_3_ clusters appeared within 2–3 min post treatment as depicted in the animation (Online Resource 1). Over the whole time-course, PIP_3_ clusters were dynamic, with some disappearing upon LecA internalization (blue arrows in Fig. 5).

**Fig. 6:**
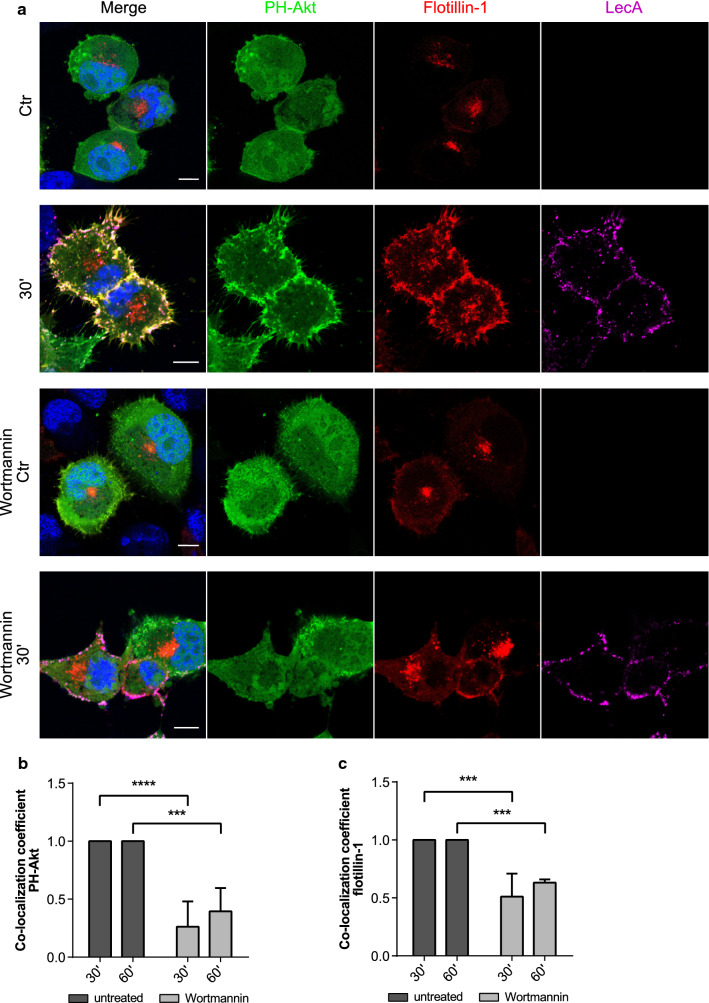
PI3-kinase inhibition diminishes PIP_3_ clustering and recruitment of flotillins upon LecA stimulation. **a** Confocal microscopy images of PH-Akt-GFP and flotillin-1-mCherry expressing H1299 cells exposed to fluorescent LecA. Lower two panels: cells were pre-treated with 100 nM Wortmannin to inhibit PI3-kinase activity. Scale bar: 10 μm. **b** Co-localization of PH-Akt-GFP and LecA is depicted as fold change of Mander’s co-localization coefficient normalized to the untreated conditions. **c** Fold change of Mander’s co-localization coefficient quantified between the fluorescence signals of flotillin-1-mCherry and LecA in comparison to the untreated conditions. For all panels: bars display mean values of three biological replicates, error bars represent SD **p < 0.01, ***p < 0.001, ****p < 0.0001 (two-way ANOVA and Tukey’s multiple comparisons tests)

These observations encouraged us to study a potential link between the PI3-kinase and flotillins by co-transfection of H1299 cells with PH-Akt-GFP and flotillin-1-mCherry (Fig. 6). Strikingly, a clear co-localization of all three proteins (PH-Akt, flotillin-1 and LecA) was observed after 30 min of stimulation (Fig. 6a). As described before, flotillin-1 was recruited to the plasma membrane and accumulated at LecA nucleation domains. Addition of Wortmannin, a potent inhibitor of PI3-kinase activity [61], disrupted the co-localization of PH-Akt-GFP and LecA (Fig. 6b) and lowered the number of PH-Akt-GFP clusters. Interestingly, this reduced the co-localization of flotillin-1-mCherry and LecA (Fig. 6c). Moreover, Wortmannin-treatment abrogated recruitment of flotillin-1 to the plasma membrane and diminished uptake of LecA. Similar results were obtained for a co-transfection of PH-Akt-GFP and flotillin-2 (Fig. S8). Taken together, we propose that the LecA-induced recruitment of flotillins to the plasma membrane depends on PI3-kinase activity leading to phosphorylation of PIP_2_. The induced processes are important for efficient internalization of LecA.

### CD59 and flotillins assist *P. aeruginosa* invasion into H1299 cells

**Fig. 7:**
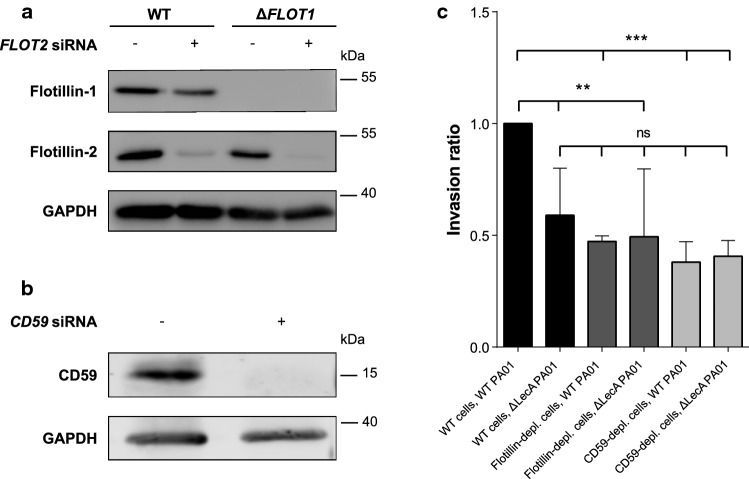
Flotillins and CD59 promote cellular invasion of *P. aeruginosa* in conjunction with LecA. **a** CRISPR-Cas9 knockout of *FLOT1* and siRNA transfection of *FLOT2* depleted H1299 cells almost completely of flotillins. Note, that protein levels of flotillins are interdependent. **b** CD59 was efficiently knocked-down by siRNA transfection of WT H1299 cells. **c** Invasion assay of WT, flotillin- and CD59-depleted H1299 cells using WT PAO1 and Δ*LecA* mutant PAO1 strains demonstrated the impact of flotillins and CD59 on PAO1 invasion. Invasion ratio was normalized to WT cells and WT PAO1. Bars display mean values of at least three biological replicates, error bars represent SD, **p < 0.01. ***p < 0.001 (one-way ANOVA and Dunnett’s multiple comparisons test)

LecA is crucial for the pathogenicity of PA [5–8]. Here, we identified the proteins CD59, flotillin-1 and -2, the Src family kinases, Rac1 and PIP_3_ as components of the plasma membrane domain for LecA-induced signaling and entry. Src kinases, small GTPases and PIPs are host cell factors with an established role in PA infection [16, 62, 63]. CD59 and flotillins, however, have not been studied in this context yet and we, therefore, aimed to understand the impact of these newly identified proteins on host cell invasion of PA. The invasion efficiency of PAO1 was analyzed with respect to the presence of flotillins and CD59 in H1299 cells (Fig. 7). To do so, we first created a CRISPR-Cas9 knockout model of *FLOT1* (Δ*FLOT1*) in H1299 cells (Fig. S9). To negate the effects of flotillin-2, we additionally silenced *FLOT2* expression by transfection of Δ*FLOT1* cells with *FLOT2* siRNA (Fig. 7a). Here, the interdependency of the two flotillins could be highlighted in an immunoblot of WT and Δ*FLOT1* lysates additionally subjected to flotillin-2 silencing: flotillin-1 levels decreased in WT cells transfected with *FLOT2* siRNA, while less flotillin-2 was detected in Δ*FLOT1* cells as compared to H1299 WT. Additionally, the knockdown of flotillin-2 was more efficient in Δ*FLOT1* cells. We were, therefore, able to conduct the experiments in H1299 cells almost entirely depleted of flotillins. Similarly, expression of CD59 was silenced by *CD59* siRNA transfection of H1299 WT cells (Fig. 7b).

Strikingly, the experiments demonstrated a reduction in PAO1 invasiveness by about 50% or 60% in H1299 cells depleted of flotillins or CD59, respectively (Fig. 7c). These observations clearly point towards a promotional role of the two proteins in PAO1 invasion. Similarly, infection of H1299 WT host cells with a Δ*LecA* PAO1 mutant strain reduced the invasion efficiency to comparable levels of WT PAO1 invasion into CD59- or flotillin-depleted cells. Interestingly, infection of CD59- or flotillin-depleted cells with Δ*LecA* PAO1 did not further lower the invasiveness, which suggests that LecA, CD59 and flotillins act in the same pathway. Our approach, furthermore, demonstrates the successful transfer of results obtained by studying a single bacterial virulence factor, namely LecA, to the complete bacterium—a process that facilitates the identification of key players in infection.

## Discussion

Membrane processes related to the uptake of PA into host cells are only beginning to be elucidated, in part because this bacterium is mostly considered as an opportunistic extracellular pathogen [64]. However, at certain sites of colonization, for example in the lungs, invasive strains of PA cause severe and chronic infections. So far, PIPs [63], Src family kinases [62, 65], caveolins [66, 67], the actin cytoskeleton and the microtubule network [8, 68, 69] are host cell factors implicated in the internalization process of PA. Here, we demonstrate that the flotillin family and the GPI-anchored protein CD59 (one of the cargo proteins of flotillin-assisted endocytosis) play a significant role in PAO1 invasion into lung epithelial cells. Flotillins are evolutionary conserved and ubiquitously expressed proteins with particularly high expression levels in heart, brain and lungs [70]. Nevertheless, their cellular functions are incompletely understood and their role in endocytosis is divisive. On one hand, Glebov and colleagues investigated the uptake of CD59 and GM1, the cholera toxin receptor, and demonstrated a requirement for flotillins and dynamin but not for clathrin in endocytosis of these cargos [40]. On the other hand, several studies suggest that flotillins might work hand-in-hand with clathrin-mediated endocytosis by clustering of cargos prior to their internalization via clathrin [41, 71–73]. Similarly, the interaction between flotillins and caveolins is controversially discussed. Various studies describe the segregation of flotillins and caveolins into distinct membrane domains [40, 74–77]. However, an interaction between the two proteins was demonstrated in other publications [78–81] including the identification of a functional link during insulin-induced glucose transporter type-4 trafficking to the plasma membrane [82]. Furthermore, flotillins coordinate cargo sorting, recycling and trafficking of several toxins without an influence on their uptake per se [83–85].

Little is known about the interplay between flotillins and pathogens. Korhonen and colleagues demonstrated a reduced intracellular growth of *Chlamydia pneumoniae* in the absence of flotillin-1 [43]. In addition, the invasion of erythrocytes by the parasite *Plasmodium falciparum* is dependent on lipid rafts and the recruitment of both flotillin proteins and CD59 to the parasitophorous vacuole [86–88]. A recent study strengthens the available data by highlighting a critical role of flotillins in *Anaplasma phagocytophilum* infection [44].

The intricacy of bacteria and the host plasma membrane complicates research on a molecular level. By reducing the complexity of the system and analyzing the interaction of a single bacterial factor, namely LecA, with the host cell plasma membrane, we gained insights into the key players of binding and uptake processes and determined important parameters for the invasiveness of *P.* *aeruginosa* strains. Interaction partners of LecA were identified by purification of proteins directly or indirectly bound by LecA-biotin using proteomic analysis. Strikingly, many detected proteins are known lipid raft components including the GPI-anchored protein CD59, the flotillin proteins and the Src family kinase Yes [89–91]. The complement regulation factor CD59 was already functionally linked to Src family kinases in studies demonstrating the recruitment and activation of trimeric G proteins and the Src kinase Lyn to CD59 clusters in the plasma membrane [89, 92]. Additionally, CD59 is a known cargo of flotillin-assisted endocytosis [40] and highlighted in several studies as an interaction partner of flotillins [74, 77].

Moreover, both flotillins can be tyrosine-phosphorylated by members of the Src family kinases and closely associate at the plasma membrane [91, 93]. As we previously demonstrated [17] and verified here, Src family kinases are activated and phosphorylated at Tyr^416^ upon LecA treatment of H1299 cells. The activation of Src kinases might eventually culminate in cytoskeletal reorganization, a prerequisite for the uptake of lectins and whole bacteria. Furthermore, we detected known endocytic players of lipid rafts in our MS screen. These include the small G protein Rac1 and cytoskeletal components like vimentin and myosin 9, of which the latter, interestingly, is regulated by flotillins [56]. Additionally, an impact of the lipid raft marker caveolin-1 on the re-localization of flotillins upon LecA stimulation was observed. Even though we did not detect caveolin-1 in our MS analysis, where we investigated early time points of LecA stimulation up to 15 min, its participation in the establishment of the flotillin-enriched membrane domain at later time points is reasonable. In accordance with the described dependence of PA invasion on lipid rafts [66, 94, 95], the here-identified factors might work together to orchestrate the LecA-triggered uptake of PAO1.

Assembly of transmembrane receptors and scaffolding proteins into specialized membrane domains is as important as the clustering of lipids on both membrane leaflets for enabling interactions with recruited proteins and signal transduction. To further characterize the LecA-induced plasma membrane domain, we analyzed the Gb3 species pulled-down together with LecA-biotin. The three most abundant Gb3 species present in H1299 cells were Gb3(d18:1/16:0), Gb3(d18:1/24:0) and Gb3(d18:1/24:1) as determined by lipid MS. These results agree with data presented in [96], where the composition of Gb3 species in several cell lines is summarized. Strikingly, the saturated Gb3 species Gb3(d18:1/16:0) and Gb3(d18:1/24:0) were enriched roughly by a factor of 1.7 in comparison to the unsaturated species Gb3(d18:1/24:1) in eluates of LecA pull-down samples. The saturation level of Gb3 ceramide tails has been shown to drastically affect lipid bilayer phases [97]. Therefore, it is unsurprising that several studies describe the critical impact of acyl chain length and degree of saturation on binding and trafficking of protein toxins [21, 26, 98, 99]. The detected preference of LecA towards saturated Gb3 species in our MS analysis suggests preferential binding to rather tightly packed membrane domains, a characteristic feature of lipid rafts. Gb3 species with little abundance in the plasma membrane in our study, e.g., hydroxylated Gb3 species, were reported to play a crucial role in the scission of StxB-induced membrane tubules [100].

Several studies investigated the binding of StxB to its receptor Gb3 in synthetic model membranes with diverse results [22, 98, 99]. Interestingly, it was shown for StxB and simian virus 40 that successful trafficking to the endoplasmic reticulum and induction of toxicity requires binding to long saturated GSLs [101–103], while cholera toxin only sorted efficiently from the plasma membrane to the Golgi network and endoplasmic reticulum by interaction with unsaturated ceramide chains of its receptor, the GSL GM1 [85]. This provides further evidence that subtle changes in the lipid bilayer influence carbohydrate exposure and consequently allow, weaken or restrict lectin binding. Furthermore, the surrounding membrane environment is critical for Gb3 receptor function [104, 105]. Depletion of cholesterol by MβCD significantly decreased levels of isolated Gb3(d18:1/16:0) and Gb3(d18:1/24:1) species. The amount of measured Gb3(d18:1/24:0) varied between the three replicates but did not change significantly. The predilection of LecA-biotin towards the saturated Gb3 species was, however, maintained.

Of note, LecA and StxB both bind to Gb3, but partially localize to different membrane domains [23] and exhibit distinct trafficking routes [106]. In our analysis, no clear difference in binding behavior to Gb3 species of LecA in comparison to StxB was observed. Therefore, we suggest that the fates of the endocytosed lectins are rather determined by the set of cellular interaction partners responsible for downstream signal transduction and cargo sorting.

**Fig. 8:**
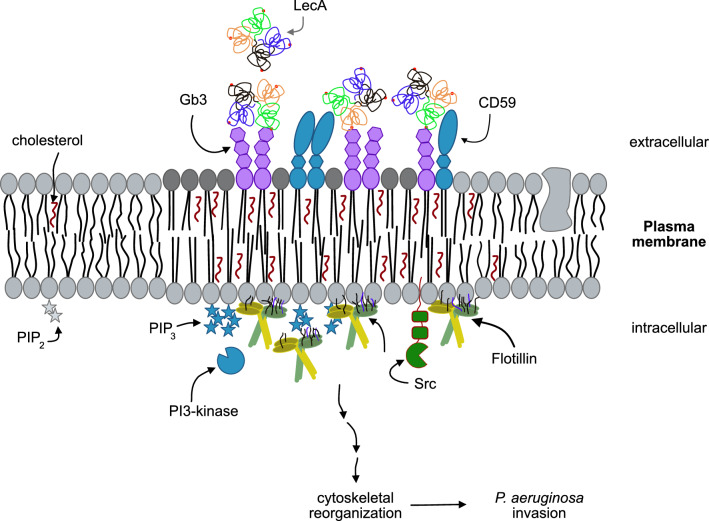
Proposed model of the interactions of LecA with the host cell plasma membrane. LecA binds to its receptor, the GSL Gb3, inducing clustering of saturated Gb3 species and recruitment of the GPI-anchored protein CD59 within the extracellular leaflet. At the intracellular leaflet PI3-kinases phosphorylate PIP_2_ to PIP_3_, which favors the recruitment of flotillins to the LecA-induced plasma membrane domain. The signal may be transduced from the extracellular to the intracellular site by transbilayer coupling between long fatty acyl chains of Gb3 and CD59 on one hand and PIPs on the other hand. Small GTPases like Rac1 and Src family kinases are known to mediate cytoskeletal reorganization. The formation of the LecA plasma membrane domain primes the host cell for an efficient uptake of PA. Components are not drawn to scale

The binding of LecA to Gb3 generates a signal at the extracellular leaflet but so far no transmembrane-spanning protein linking Gb3 with the intracellular membrane leaflet is known. How is the signal then communicated to flotillins and Src kinases? A long-standing, popular hypothesis suggests a role for fatty acids in signal transduction [19, 107]. Pinto and colleagues studied the influence of ceramide (i.e., the backbone of Gb3) structure and acyl chain length and demonstrated that long-chain ceramides induce strong alterations in the bilayer, suggesting the formation of interdigitating phases [108]. Further, GPI-anchored proteins with long saturated acyl chains can interdigitate and connect the membrane leaflets given that one of the two leaflets is immobilized [20]. A good example of transbilayer coupling through GPI-anchored proteins might be the prion protein. Clustering of the prion protein in the extracellular leaflet was suggested to influence flotillins at the intracellular leaflet through interaction with flotillin myristoyl- and palmitoyl-residues [39]. Recently, PIPs were proposed to participate in transbilayer coupling [58]. Since PIPs naturally bear a long saturated C18 acyl chain, they can reach across the bilayer and interact with long fatty acyl chains of lipids located in the extracellular leaflet. Here, we demonstrate an induction of PIP_3_ clustering upon LecA stimulation of H1299 cells. Cluster formation was crucial for flotillin recruitment to the plasma membrane. LecA-induced co-clustering of long, saturated Gb3 species and the GPI-anchored protein CD59 with PIPs might therefore enable communication across the plasma membrane, inducing intracellular processes that culminate in endocytosis. Flotillins functionally scaffold and enhance signal transduction [32, 53]. Since internalization of PA is known to require PI3-kinase and Src family kinase activity [62, 63, 65], the induction of these processes by LecA primes the cellular plasma membrane for PA uptake (Fig. 8, proposed model).

## Conclusion

In this study, we characterize the host cell plasma membrane domain to which the PA virulence factor LecA binds selectively. This membrane domain is composed of saturated Gb3 species along with the GPI-anchored protein CD59. Upon LecA binding and clustering of Gb3, intracellular flotillins are recruited and scaffold active signaling platforms including Src family kinases. The lectin-induced signal may be transmitted from the extra- to the intracellular leaflet by transbilayer coupling between long fatty acyl chains of Gb3 or CD59 and PIPs. Importantly, we discovered that flotillins and CD59 promote PA invasion into lung epithelial cells. We, therefore, propose a model in which LecA prepares and reorganizes the plasma membrane to facilitate entry of PA.

## Supplementary Information

Below is the link to the electronic supplementary material.

Supplementary file1 (EPS 723 KB)

Supplementary file2 (EPS 802 KB)

Supplementary file3 (EPS 602 KB)

Supplementary file4 (EPS 743 KB)

Supplementary file5 (EPS 612 KB)

Supplementary file6 (EPS 546 KB)

Supplementary file7 (EPS 1392 KB)

Supplementary file8 (EPS 3442 KB)

Supplementary file9 (EPS 98 KB)

Supplementary file10 (EPS 764 KB)

Supplementary file11 (EPS 848 KB)

Supplementary file12 (AVI 41313 KB)

Supplementary file13 (DOCX 19659 KB)

Supplementary file14 (DOCX 12 KB)

## Data Availability

The datasets generated and/or analyzed during the current study are available from the corresponding author on reasonable request.

## Abbreviations

ANOVA: Analysis of variance
Δ*FLOT1*: CRISPR-Cas9 knockout model of *FLOT1*
DPPS: Dulbecco’s phosphate-buffered saline
GAPDH: Glyceraldehyde 3-phosphate dehydrogenase
Gb3: Globotriaosylceramide
GPI: Glycosylphosphatidylinositol
GSL: Glycosphingolipid
IP: Immunoprecipitation
LC: Liquid chromatography
MOI: Multiplicity of infection
MS: Mass spectrometry
MβCD: Methyl-beta-cyclodextrin
PA: *Pseudomonas aeruginosa*
PI3-kinase: Phosphoinositide 3-kinase
PIP_2_: Phosphatidylinositol (4,5)-bisphosphate
PIP_3_: Phosphatidylinositol (3,4,5)-trisphosphate
PIPs: Phosphatidylinositols
PPMP: D-threo-l-phenyl-2-palmitoylamino-3-morpholino-l-propanol
RIPA: Radio-immunoprecipitation assay
RPMI: Roswell Park Memorial Institute
RT: Room temperature
SD: Standard deviation
sgRNA: Single guide RNAs
StxB: Shiga toxin B-subunit
WT: Wild type

## References

[1] WHO (2017) Global priority list of antibiotic-resistant batceria to guide research, discovery, and development of new antibiotics. WHO [Internet]. https://www.who.int/news-room/detail/27-02-2017-who-publishes-list-of-bacteria-for-which-new-antibiotics-are-urgently-needed

[2] Rossolini GM, Mantengoli E (2005). Treatment and control of severe infections caused by multiresistant* Pseudomonas aeruginosa*. Clin Microbiol Infect Suppl.

[3] Lyczak JB, Cannon CL, Pier GB (2000). Establishment of *Pseudomonas aeruginosa* infection: lessons from a versatile opportunist. Microbes Infect.

[4] Gilboa-Garber N, Sudakevitz D, Sheffi M, Sela R, Levene C (1994). Pa-I and Pa-II lectin interactions with the Abo(H) and P-blood group glycosphingolipid antigens may contribute to the broad-spectrum adherence of *Pseudomonas aeruginosa* to human tissues in secondary infections. Glycoconj J.

[5] Laughlin RS, Musch MW, Hollbrook CJ, Rocha FM, Chang EB, Alverdy JC (2000). The key role of Pseudomonas aeruginosa PA-I lectin on experimental gut-derived sepsis. Ann Surg.

[6] Diggle SP, Stacey RE, Dodd C, Cámara M, Williams P, Winzer K (2006). The galactophilic lectin, LecA, contributes to biofilm development in *Pseudomonas aeruginosa*. Environ Microbiol.

[7] Chemani C, Imberty A, De Bentzmann S, Pierre M, Wimmerová M, Guery BP, Faure K (2009). Role of LecA and LecB lectins in *Pseudomonas aeruginosa*—induced lung injury and effect of carbohydrate ligands. Infect Immun.

[8] Eierhoff T, Bastian B, Thuenauer R, Madl J, Audfray A, Aigal S, Juillot S, Rydell GE, Muller S, de Bentzmann S (2014). A lipid zipper triggers bacterial invasion. Proc Natl Acad Sci.

[9] Glick J, Garber N (1983). The intracellular localization of *Pseudomonas aeruginosa* lectins. J Gen Microbiol.

[10] Lingwood CA (1993). Verotoxins and their glycolipid receptors. Adv Lipid Res.

[11] Novoa A, Eierhoff T, Topin J, Varrot A, Barluenga S, Imberty A, Römer W, Winssinger N (2014). A LecA ligand identified from a galactoside-conjugate array inhibits host cell invasion by *Pseudomonas aeruginosa*. Angew Chemie Int Ed.

[12] Eierhoff T, Stechmann B, Römer W (2012) Pathogen and toxin entry—how pathogens and toxins induce and harness endocytotic mechanisms. In: Ceresa B (ed) Molecular regulation of endocytosis (InTech). pp 249–276. ISBN 980-953-307-015-0

[13] Aigal S, Claudinon J, Römer W (2015). Plasma membrane reorganization: a glycolipid gateway for microbes. Biochim Biophys Acta Mol Cell Res.

[14] Feller SM (2001). CrK family adaptors-signalling complex formation and biological roles. Oncogene.

[15] Antoku S, Mayer BJ (2009). Distinct roles for Crk adaptor isoforms in actin reorganization induced by extracellular signals. J Cell Sci.

[16] Pielage JF, Powell KR, Kalman D, Engel JN (2008). RNAi screen reveals an Abl kinase-dependent host cell pathway involved in *Pseudomonas aeruginosa* internalization. PLoS Pathog.

[17] Zheng S, Eierhoff T, Aigal S, Brandel A, Thuenauer R, de Bentzmann S, Imberty A, Römer W (2017). The* Pseudomonas aeruginosa* lectin LecA triggers host cell signalling by glycosphingolipid-dependent phosphorylation of the adaptor protein CrkII. Biochim Biophys Acta Mol Cell Res.

[18] Mehlhorn IE, Florio E, Barber KR, Lordo C, Grant CWM (1988). Evidence that trans-bilayer interdigitation of glycosphingolipid long chain fatty acids may be a general phenomenon. BBA Biomembr.

[19] Boggs JM, Koshy KM (1994). Do the long fatty acid chains of sphingolipids interdigitate across the center of a bilayer of shorter chain symmetric phospholipids?. Biochim Biophys Acta.

[20] Raghupathy R, Anilkumar AA, Polley A, Singh PP, Yadav M, Johnson C, Suryawanshi S, Saikam V, Sawant SD, Panda A (2015). Transbilayer lipid interactions mediate nanoclustering of lipid-anchored proteins. Cell.

[21] Kiarash A, Boyd B, Lingwood CA (1994). Glycosphingolipid receptor function is modified by fatty acid content. J Biol Chem.

[22] Schütte OM, Ries A, Orth A, Patalag LJ, Römer W, Steinem C, Werz DB (2014). Influence of Gb3 glycosphingolipids differing in their fatty acid chain on the phase behaviour of solid supported membranes: chemical syntheses and impact of Shiga toxin binding. Chem Sci.

[23] Schubert T, Sych T, Madl J, Xu M, Omidvar R, Patalag LJ, Ries A, Kettelhoit K, Brandel A, Mely Y (2020). Differential recognition of lipid domains by two Gb3-binding lectins. Sci Rep.

[24] Simons K, Van Meer G (1988). Lipid sorting in epithelial cells. Biochemistry.

[25] Brown DA, Rose JK (1992). Sorting of GPI-anchored proteins to glycolipid-enriched membrane subdomains during transport to the apical cell surface. Cell.

[26] Lingwood D, Simons K (2010). Lipid rafts as a membrane-organizing principle. Science.

[27] Simons K, Ikonen E (1997). Functional rafts in cell membranes. Nature.

[28] Kasahara K, Sanai Y (1999). Possible roles of glycosphingolipids in lipid rafts. Biophys Chem.

[29] Doherty GJ, McMahon HT (2009). Mechanisms of endocytosis. Annu Rev Biochem.

[30] Elkin SR, Lakoduk AM, Schmid SL (2016). Endocytic pathways and endosomal trafficking: a primer. Wien Med Wochenschr.

[31] Bauer M, Pelkmans L (2006). A new paradigm for membrane-organizing and -shaping scaffolds. FEBS Lett.

[32] Langhorst MF, Reuter A, Stuermer CAO (2005). Scaffolding microdomains and beyond: the function of reggie/flotillin proteins. Cell Mol Life Sci.

[33] Neumann-Glesen C, Falkenbach B, Beicht P, Claasen S, Lüers G, Stuermer CAO, Herzog V, Tikkanen R (2004). Membrane and raft association of reggie-1/flotillin-2: Role of myristoylation, palmitoylation and oligomerization and induction of filopodia by overexpression. Biochem J.

[34] Stuermer CAO (2011). Microdomain-forming proteins and the role of the reggies/flotillins during axon regeneration in zebrafish. Biochim Biophys Acta Mol Basis Dis.

[35] Langhorst MF, Solis GP, Hannbeck S, Plattner H, Stuermer CAO (2007). Linking membrane microdomains to the cytoskeleton: regulation of the lateral mobility of reggie-1/flotillin-2 by interaction with actin. FEBS Lett.

[36] Otto GP, Nichols BJ (2011). The roles of flotillin microdomains— endocytosis and beyond. J Cell Sci.

[37] Kurrle N, Völlner F, Eming R, Hertl M, Banning A, Tikkanen R (2013). Flotillins directly interact with γ-catenin and regulate epithelial cell-cell adhesion. PLoS ONE.

[38] Kioka N, Ueda K, Amachi T (2002). Vinexin, CAP/ponsin, ArgBP2: a novel adaptor protein family regulating cytoskeletal organization and signal transduction. Cell Struct Funct.

[39] Stuermer CAO (2010). The reggie/flotillin connection to growth. Trends Cell Biol.

[40] Glebov OO, Bright NA, Nichols BJ (2006). Flotillin-1 defines a clathrin-independent endocytic pathway in mammalian cells. Nat Cell Biol.

[41] Schneider A, Rajendran L, Honsho M, Gralle M, Donnert G, Wouters F, Hell SW, Simons M (2008). Flotillin-dependent clustering of the amyloid precursor protein regulates its endocytosis and amyloidogenic processing in neurons. J Neurosci.

[42] Cremona ML, Matthies HJG, Pau K, Bowton E, Speed N, Lute BJ, Anderson M, Sen N, Robertson SD, Vaughan RA (2011). Flotillin-1 is essential for PKC-triggered endocytosis and membrane microdomain localization of DAT. Nat Neurosci.

[43] Korhonen JT, Puolakkainen M, Häivälä R, Penttilä T, Haveri A, Markkula E, Lahesmaa R (2012). Flotillin-1 (reggie-2) contributes to *Chlamydia pneumoniae* growth and is associated with bacterial inclusion. Infect Immun.

[44] Xiong Q, Lin M, Huang W, Rikihisa Y (2019). Infection by anaplasma phagocytophilum requires recruitment of low-density lipoprotein cholesterol by flotillins. MBio.

[45] Abe A, Inokuchi J, Jimbo M, Shimeno H, Nagamatsu A, Shayman JA, Shukla GS, Radin NS (1992). Improved inhibitors of glucosylceramide synthase. J Biol Chem.

[46] Sapcariu SC, Kanashova T, Weindl D, Ghelfi J, Dittmar G, Hiller K (2014). Simultaneous extraction of proteins and metabolites from cells in culture. MethodsX.

[47] Lagies S, Schlimpert M, Neumann S, Wäldin A, Kammerer B, Borner C, Peintner L (2020). Cells grown in three-dimensional spheroids mirror in vivo metabolic response of epithelial cells. Commun Biol.

[48] Ishihama Y, Oda Y, Tabata T, Sato T, Nagasu T, Rappsilber J, Mann M (2005). Exponentially modified protein abundance index (emPAI) for estimation of absolute protein amount in proteomics by the number of sequenced peptides per protein. Mol Cell Proteomics.

[49] Ran FA, Hsu PD, Wright J, Agarwala V, Scott DA, Zhang F (2013). Genome engineering using the CRISPR-Cas9 system. Nat Protoc.

[50] Holloway BW (1955). Genetic recombination in *Pseudomonas aeruginosa*. J Gen Microbiol.

[51] Falguières T, Mallard F, Baron C, Hanau D, Lingwood C, Goud B, Salamero J, Johannes L (2001). Targeting of Shiga toxin B-subunit to retrograde transport route in association with detergent-resistant membranes. Mol Biol Cell.

[52] Lyczak JB, Cannon CL, Pier GB (2002). Lung infections associated with cystic fibrosis. Clin Microbiol Rev.

[53] Meister M, Tikkanen R (2014). Endocytic trafficking of membrane-bound cargo: a flotillin point of view. Membranes (Basel).

[54] Raucher D, Stauffer T, Chen W, Shen K, Guo S, York JD, Sheetz MP, Meyer T (2000). Phosphatidylinositol 4,5-bisphosphate functions as a second messenger that regulates cytoskeleton-plasma membrane adhesion. Cell.

[55] Head JA, Jiang D, Li M, Zorn LJ, Schaefer EM, Parsons JT, Weed SA (2003). Cortactin tyrosine phosphorylation requires Rac1 activity and association with the cortical actin cytoskeleton. Mol Biol Cell.

[56] Ludwig A, Otto GP, Riento K, Hams E, Fallon PG, Nichols BJ (2010). Flotillin microdomains interact with the cortical cytoskeleton to control uropod formation and neutrophil recruitment. J Cell Biol.

[57] Michell RH (2008). Inositol derivatives: evolution and functions. Nat Rev Mol Cell Biol.

[58] Sengupta P, Seo AY, Pasolli HA, Song YE, Johnson MC, Lippincott-Schwartz J (2019). A lipid-based partitioning mechanism for selective incorporation of proteins into membranes of HIV particles. Nat Cell Biol.

[59] Meili R, Ellsworth C, Lee S, Reddy TBK, Ma H, Firtel RA (1999). Chemoattractant-mediated transient activation and membrane localization of Akt/PKB is required for efficient chemotaxis to cAMP in Dictyostelium. EMBO J.

[60] Servant G, Weiner OD, Herzmark P, Balla T, Sedat JW, Bourne HR (2000). Polarization of chemoattractant receptor signaling during neutrophil chemotaxis. Science.

[61] Powis G, Bonjouklian R, Berggren MM, Gallegos A, Abraham R, Ashendel C, Zalkow L, Matter WF, Dodge J, Grindey G (1994). Wortmannin, a potent and selective inhibitor of phosphatidylinositol-3-kinase. Cancer Res.

[62] Esen M, Grassmé H, Riethmüller J, Riehle A, Fassbender K, Gulbins E (2001). Invasion of human epithelial cells by *Pseudomonas aeruginosa* involves Src-like tyrosine kinases p60Src and p59Fyn. Infect Immun.

[63] Kierbel A, Gassama-Diagne A, Mostov K, Engel JN (2005). The phosphoinositol-3-kinase–protein kinase B/Akt pathway is critical for *Pseudomonas aeruginosa* strain PAK internalization. Mol Biol Cell.

[64] Lovewell RR, Patankar YR, Berwin B (2014). Mechanisms of phagocytosis and host clearance of *Pseudomonas aeruginosa*. Am J Physiol Lung Cell Mol Physiol.

[65] Kannan S, Audet A, Huang H, Chen L, Wu M (2008). Cholesterol-rich membrane rafts and Lyn are involved in phagocytosis during *Pseudomonas aeruginosa* infection. J Immunol.

[66] Zaas DW, Duncan MJ, Li G, Wright JR, Abraham SN (2005). Pseudomonas invasion of type I pneumocytes is dependent on the expression and phosphorylation of caveolin-2. J Biol Chem.

[67] Gadjeva M, Paradis-Bleau C, Priebe GP, Fichorova R, Pier GB (2010). Caveolin-1 modifies the immunity to *Pseudomonas aeruginosa*. J Immunol.

[68] Evans DJ, Frank DW, Finck-Barbançon V, Wu C, Fleiszig SMJ (1998). *Pseudomonas aeruginosa* invasion and cytotoxicity are independent events, both of which involve protein tyrosine kinase activity. Infect Immun.

[69] Sana TG, Baumann C, Merdes A, Soscia C, Rattei T, Hachani A, Jones C, Bennett KL, Filloux A, Superti-Furga G (2015). Internalization of *Pseudomonas aeruginosa* strain PAO1 into epithelial cells is promoted by interaction of a T6SS effector with the microtubule network. MBio.

[70] Banning A, Kurrle N, Meister M, Tikkanen R (2014). Flotillins in receptor tyrosine kinase signaling and cancer. Cells.

[71] Sorkina T, Hoover BR, Zahniser NR, Sorkin A (2005). Constitutive and protein kinase C-induced internalization of the dopamine transporter is mediated by a clathrin-dependent mechanism. Traffic.

[72] Ge L, Qi W, Wang LJ, Miao HH, Qu YX, Li BL, Song BL (2011). Flotillins play an essential role in Niemann-Pick C1-like 1-mediated cholesterol uptake. Proc Natl Acad Sci USA.

[73] Amaddii M, Meister M, Banning A, Tomasovic A, Mooz J, Rajalingam K, Tikkanen R (2012). Flotillin-1/Reggie-2 protein plays dual role in activation of receptor-tyrosine kinase/mitogen-activated protein kinase signaling. J Biol Chem.

[74] Frick M, Bright NA, Riento K, Bray A, Merrified C, Nichols BJ (2007). Coassembly of flotillins induces formation of membrane microdomains, membrane curvature, and vesicle budding. Curr Biol.

[75] Roitbak T, Surviladze Z, Tikkanen R, Wandinger-Ness A (2005). A polycystin multiprotein complex constitutes a cholesterol-containing signalling microdomain in human kidney epithelia. Biochem J.

[76] Mellgren RL (2008). Detergent-resistant membrane subfractions containing proteins of plasma membrane, mitochondrial, and internal membrane origins. J Biochem Biophys Methods.

[77] Aït-Slimane T, Galmes R, Trugnan G, Maurice M (2009). Basolateral internalization of GPI-anchored proteins occurs via a clathrin-independent flotillin-dependent pathway in polarized hepatic cells. Mol Biol Cell.

[78] Bickel PE, Scherer PE, Schnitzer JE, Oh P, Lisanti MP, Lodish HF (1997). Flotillin and epidermal surface antigen define a new family of caveolae—associated integral membrane proteins. J Biol Chem.

[79] Volonté D, Galbiati F, Li S, Nishiyama K, Okamoto T, Lisanti MP (1999). Flotillins/cavatellins are differentially expressed in cells and tissues and form a hetero-oligomeric complex with caveolins in vivo: Characterization and epitope-mapping of a novel flotillin-1 monoclonal antibody probe. J Biol Chem.

[80] Baumann CA, Ribon V, Kanzaki M, Thurmond DC, Mora S, Shigematsu S, Bickel PE, Pessin JE, Saltiel AR (2000). CAP defines a second signalling pathway required for insulin-stimulated glucose transport. Nature.

[81] Vassilieva EV, Ivanov AI, Nusrat A (2009). Flotillin-1 stabilizes caveolin-1 in intestinal epithelial cells. Biochem Biophys Res Commun.

[82] Fecchi K, Volonte D, Hezel MP, Schmeck K, Galbiati F (2006). Spatial and temporal regulation of GLUT4 translocation by flotillin-1 and caveolin-3 in skeletal muscle cells. FASEB J.

[83] Pust S, Dyve AB, Torgersen ML, Van Deurs B, Sandvig K (2010). Interplay between toxin transport and flotillin localization. PLoS ONE.

[84] Saslowsky DE, Cho JA, Chinnapen H, Massol RH, Chinnapen DJF, Wagner JS, De Luca HE, Kam W, Paw BH, Lencer WI (2010). Intoxication of zebrafish and mammalian cells by cholera toxin depends on the flotillin/reggie proteins but not Derlin-1 or -2. J Clin Invest.

[85] Chinnapen DJF, Hsieh WT, te Welscher YM, Saslowsky DE, Kaoutzani L, Brandsma E, D’Auria L, Park H, Wagner JS, Drake KR (2012). Lipid sorting by ceramide structure from plasma membrane to ER for the cholera toxin receptor ganglioside GM1. Dev Cell.

[86] Lauer S, VanWye J, Harrison T, McManus H, Samuel BU, Hiller NL, Mohandas N, Haldar K (2000). Vacuolar uptake of host components, and a role for cholesterol and sphingomyelin in malarial infection. EMBO J.

[87] Samuel BU, Mohandas N, Harrison T, McManus H, Rosse W, Reid M, Haldar K (2001). The role of cholesterol and glycosylphosphatidylinositol-anchored proteins of erythrocyte rafts in regulating raft protein content and malarial infection. J Biol Chem.

[88] Murphy SC, Samuel BU, Harrison T, Speicher KD, Speicher DW, Reid ME, Prohaska R, Low PS, Tanner MJ, Mohandas N (2004). Erythrocyte detergent-resistant membrane proteins: their characterization and selective uptake during malarial infection. Blood.

[89] Štefanová I, Hořejší V, Ansotegui IJ, Knapp W, Stockinger H (1991). GPI-anchored cell-surface molecules complexed to protein tyrosine kinases. Science.

[90] Lang DM, Lommel S, Jung M, Ankerhold R, Petrausch B, Laessing U, Wiechers MF, Plattner H, Stuermer CAO (1998). Identification of reggie-1 and reggie-2 as plasmamembrane-associated proteins which cocluster with activated GPI-anchored cell adhesion molecules in non-caveolar micropatches in neurons. J Neurobiol.

[91] Stuermer CAO, Lang DM, Kirsch F, Wiechers MF, Deininger S-O, Plattner H (2001). Glycosylphosphatidyl inositiol-anchored proteins and fyn kinase assemble in noncaveolar plasma membrane microdomains defined by reggie-1 and -2. Mol Biol Cell.

[92] Suzuki KGN, Fujiwara TK, Sanematsu F, Iino R, Edidin M, Kusumi A (2007). GPI-anchored receptor clusters transiently recruit Lyn and Gα for temporary cluster immobilization and Lyn activation: single-molecule tracking study 1. J Cell Biol.

[93] Neumann-Glesen C, Fernow I, Amaddii M, Tikkanen R (2007). Role of EGF-induced tyrosine phosphorylation of reggie-1/flotillin-2 in cell spreading and signaling to the actin cytoskeleton. J Cell Sci.

[94] Schiumarini D, Loberto N, Mancini G, Bassi R, Giussani P, Chiricozzi E, Samarani M, Munari S, Tamanini A, Cabrini G (2017). Evidence for the involvement of lipid rafts and plasma membrane sphingolipid hydrolases in *Pseudomonas aeruginosa* infection of cystic fibrosis bronchial epithelial cells. Mediators Inflamm.

[95] Yamamoto N, Yamamoto N, Petroll MW, Cavanagh HD, Jester JV (2005). Internalization of *Pseudomonas aeruginosa* is mediated by lipid rafts in contact lens-wearing rabbit and cultured human corneal epithelial cells. Investig Ophthalmol Vis Sci.

[96] Sandvig K, Bergan J, Kavaliauskiene S, Skotland T (2014). Lipid requirements for entry of protein toxins into cells. Prog Lipid Res.

[97] Pezeshkian W, Chaban VV, Johannes L, Shillcock J, Ipsen JH, Khandelia H (2015). The effects of globotriaosylceramide tail saturation level on bilayer phases. Soft Matter.

[98] Römer W, Berland L, Chambon V, Gaus K, Windschiegl B, Tenza D, Aly MRE, Fraisier V, Florent J-C, Perrais D (2007). Shiga toxin induces tubular membrane invaginations for its uptake into cells. Nature.

[99] Windschiegl B, Orth A, Römer W, Berland L, Stechmann B, Bassreau P, Johannes L, Steinem C (2009). Lipid reorganization induced by Shiga toxin clustering on planar membranes. PLoS ONE.

[100] Römer W, Pontani LL, Sorre B, Rentero C, Berland L, Chambon V, Lamaze C, Bassereau P, Sykes C, Gaus K (2010). Actin dynamics drive membrane reorganization and scission in clathrin-independent endocytosis. Cell.

[101] Sandvig K, Ryd M, Garred Ø, Schweda E, Holm PK, Van Deurs B (1994). Retrograde transport from the Golgi complex to the ER of both Shiga toxin and the nontoxic Shiga B-fragment is regulated by butyric acid and cAMP. J Cell Biol.

[102] Raa H, Grimmer S, Schwudke D, Bergan J, Wälchli S, Skotland T, Shevchenko A, Sandvig K (2009). Glycosphingolipid requirements for endosome-to-Golgi transport of Shiga toxin. Traffic.

[103] Ewers H, Römer W, Smith AE, Bacia K, Dmitrieff S, Chai W, Mancini R, Kartenbeck J, Chambon V, Berland L (2010). GM1 structure determines SV40-induced membrane invagination and infection. Nat Cell Biol.

[104] Smith DC, Sillence DJ, Falguières T, Jarvis RM, Johannes L, Lord JM, Platt FM, Roberts LM (2006). The association of Shiga-like toxin with detergent- resistant membranes is modulated by glucosylceramide and is an essential requirement in the endoplasmic reticulum for a cytotoxic effect. Mol Biol Cell.

[105] Lingwood CA, Binnington B, Manis A, Branch DR (2010). Globotriaosyl ceramide receptor function—where membrane structure and pathology intersect. FEBS Lett.

[106] Müller SK, Wilhelm I, Schubert T, Zittlau K, Imberty A, Madl J, Eierhoff T, Thuenauer R, Römer W (2017). Gb3-binding lectins as potential carriers for transcellular drug delivery. Expert Opin Drug Deliv.

[107] Huang C (1990). Mixed-chain phospholipids and interdigitated bilayer systems. Klin Wochenschr.

[108] Pinto SN, Silva LC, Futerman AH, Prieto M (2011). Effect of ceramide structure on membrane biophysical properties: the role of acyl chain length and unsaturation. Biochim Biophys Acta Biomembr.

