# Supplementary material for: The Gb3-enriched CD59/flotillin plasma membrane domain regulates host cell invasion by Pseudomonas aeruginosa
Source: Cell Mol Life Sci. 2021 Feb 8;78(7):3637–56. doi: 10.1007/s00018-021-03766-1 (PMC8038999; doi:10.1007/s00018-021-03766-1)
Supplement: Supplementary file 13 — Supplementary file13 (DOCX 19659 KB) [file 18_2021_3766_MOESM13_ESM.docx]

The Gb3-enriched CD59/flotillin plasma membrane domain regulates host cell invasion by *Pseudomonas aeruginosa*

Annette Brandel^1,2,3^, Sahaja Aigal^1,2,§,&^, Simon Lagies^1,4,5,&^, Manuel Schlimpert^1,4,5^, Ana Valeria Meléndez^1,2,3,5^, Maokai Xu^1,2,3^, Anika Lehmann^1,2,3^, Daniel Hummel^1,2,6^, Daniel Fisch^1,2,7,8^, Josef Madl^1,2,#^, Thorsten Eierhoff^1,2,9^, Bernd Kammerer^2,4,5^, Winfried Römer^1,2,3,5^

*^1^* *Faculty of Biology, University of Freiburg, Schänzlestraße 1, 79104, Freiburg, Germany*

*^2^* *BIOSS - Centre for Biological Signalling Studies, University of Freiburg, Schänzlestraße 18, 79104 Freiburg, Germany*

*^3^ CIBSS - Centre for Integrative Biological Signalling Studies, University of Freiburg, Schänzlestraße 18, 79104 Freiburg, Germany*

*^4^ Center for Biological Systems Analysis, University of Freiburg, Habsburgerstraße 49, 79104 Freiburg, Germany*

*^5^* *Spemann Graduate School of Biology and Medicine, University of Freiburg, Freiburg, Germany*

*^6^* *Department of Biochemistry, University of Geneva, 30 Quai Ernest-Ansermet, 1211 Geneva, Switzerland*

*^7^ Host-Toxoplasma Interaction Laboratory, The Francis Crick Institute, London, UK*

*^8^ MRC Centre for Molecular Bacteriology & Infection, Department of Infectious Disease, Imperial College London, London, UK*

*^9^* *Clinic for Vascular and Endovascular Surgery, University Hospital Münster, Albert Schweitzer Campus 1, 48149 Münster, Germany*

*^#^ Current address: Institute for Experimental Cardiovascular Medicine, University Heart Center Freiburg - Bad Krozingen, and Medical Faculty, University of Freiburg, 79110 Freiburg, Germany*

*^§^ Current address: Institute of Medical Microbiology, Virology and Hygiene, University Medical Center Hamburg-Eppendorf, Martinistraße 52, 20246 Hamburg, Germany*

*^&^* These authors contributed equally.

To whom correspondence should be addressed: winfried.roemer@bioss.uni-freiburg.de

# Supplementary Figures

### **Fig. S1** Experimental design of the study. **a** H1299 cells were incubated with biotinylated LecA for 5 and 15 min and lysed with a medium-harsh lysis buffer. The LecA-bound membrane fragments were isolated by streptavidin-coated beads and subsequently eluted using two different techniques. Depending on the target of interest, lipids or proteins were extracted from beads by methanol/water/chloroform or trypsin, respectively. After separation and purification, samples were analyzed by MS. **b** Pull-down efficiency tested by immunoblot analysis after several rounds of optimization.

**Fig. S2** StxB demonstrates a predilection for saturated Gb3 species. **a** Pulled-down Gb3 species (normalized to internal standard, input values and Gb3(d18:1/16:0)) of StxB-biotin demonstrated a preference for saturated over unsaturated Gb3 species. Significantly less Gb3(d18:1/16:0) and Gb3(d18:1/24:1) was pulled-down in MβCD-treated cells stimulated with StxB-biotin. The Gb3 species Gb3(d18:1/24:0) seemed less affected by MβCD treatment. Still, the unsaturated species Gb3(d18:1/24:1) was pulled-down least. **b** Pulled-down Gb3 species of LecA- and StxB-biotin normalized to input levels but not to Gb3(d18:1/16:0) (in contrast to Figs. 1b and S2a). Both lectins preferred saturated over unsaturated Gb3 species. **c,d** The pulled-down Gb3 species Gb3(d18:1/18:0), Gb3(d18:1/18:1), Gb3(d18:1/22:0) and Gb3(d18:1/22:1) of LecA- and StxB-biotin. Values were **c** normalized or **d** not normalized to Gb3(d18:1/16:0). Also here, a preference for saturated species was visible. Remember, these Gb3 species are present in very low amounts within the plasma membrane of H1299 cells (Fig. 1a). **e** Analysis of input lysates displayed a significant depletion of cholesterol upon MβCD treatment (10 mM). For all panels: Bars display mean values of three biological replicates, error bars represent SD, *p<0.05, **p<0.01, p<****<0.0001 (For **a**, **b**, **c**, **d**: two-way ANOVA and Tukey’s multiple comparisons tests; **e**: two-tailed unpaired t test).

­­

**Fig. S3** Heat-map of significantly (ANOVA) changed lipids between pull-downs of unstimulated (ctr), LecA- and StxB-treated H1299 WT cells. Lipids are normalized to the internal standard and input levels. Range-scaled z-scores are displayed. FFA: free fatty acid; PC: phosphatidylcholine; SM: sphingomyelin, SM(34:0) and SM(42:2) indicate total carbon chain lengths of 34 or 42, and no or two unsaturated double bonds, respectively; PE: phosphatidylethanolamine. N=3.

###

**Fig. S4** Flotillins are recruited to the plasma membrane upon LecA stimulation. **a** Fluorescence co-localization studies of flotillin-2 (red) and LecA (green) after 60 min of lectin stimulation. Nuclei were counterstained by DAPI. Framed areas were magnified. White arrows point at co-localization events at the plasma membrane, asterisks at perinuclear co-localization. Scale bar: 10 μm. **b** Co-localization between LecA and flotillin-2 was quantified by Mander’s co-localization coefficient. Bars display mean values of three biological replicates, error bars represent SD, ***p<0.001, p<****<0.0001 (one-way ANOVA and Dunnett’s multiple comparisons test). **c** Pull-down of LecA-biotin resulted in a time-dependent enrichment of flotillin-2.

### ****

### **Fig. S5** LecA does not directly bind to flotillin-1 or CD59. **a** WT H1299 lysates were subjected to SDS-PAGE and immunoblotting. The membrane was subsequently incubated with LecA-biotin for 60 min, followed by treatment with streptavidin-800 (green). Lectin blot shows proteins directly bound by LecA-biotin (via streptavidin). **b** Flotillin-1 co-immunoprecipitation of LecA-stimulated WT and PPMP-treated H1299 cells was conducted. Subsequently, lectin blot analysis of the obtained lysates was performed. No direct binding of LecA (via streptavidin, green) to flotillin-1 (red) in the eluates was detectable. **c** CD59 co-immunoprecipitation of LecA-stimulated WT and PPMP-treated H1299 cells was performed and lysates were subjected to lectin blot analysis. No direct binding of LecA (via streptavidin, green) to CD59 (red) in the eluates was observed.

**Fig. S6** LecA partially co-localizes with caveolin-1, but not as pronounced as with flotillin-1. **a** Caveolin-1 staining in unstimulated H1299 cells. Nuclei were counterstained by DAPI. Scale bar: 10 μm. **b** The co-localizations between LecA and flotillin-1 as well as between LecA and caveolin-1 were qu­antified by Mander’s co-localization coefficient. Bars display mean values of at least three biological replicates, error bars represent SD. **c** Fluorescence co-localization studies of caveolin-1 (red) and LecA (green) after 90 min of LecA stimulation. Nuclei were counterstained by DAPI. Framed area was magnified. White arrow points at co-localization event at the plasma membrane, asterisks indicate perinuclear localization of LecA only marginally co-localizing with caveolin-1. Scale bar: 10 μm. KD: Knock-down.


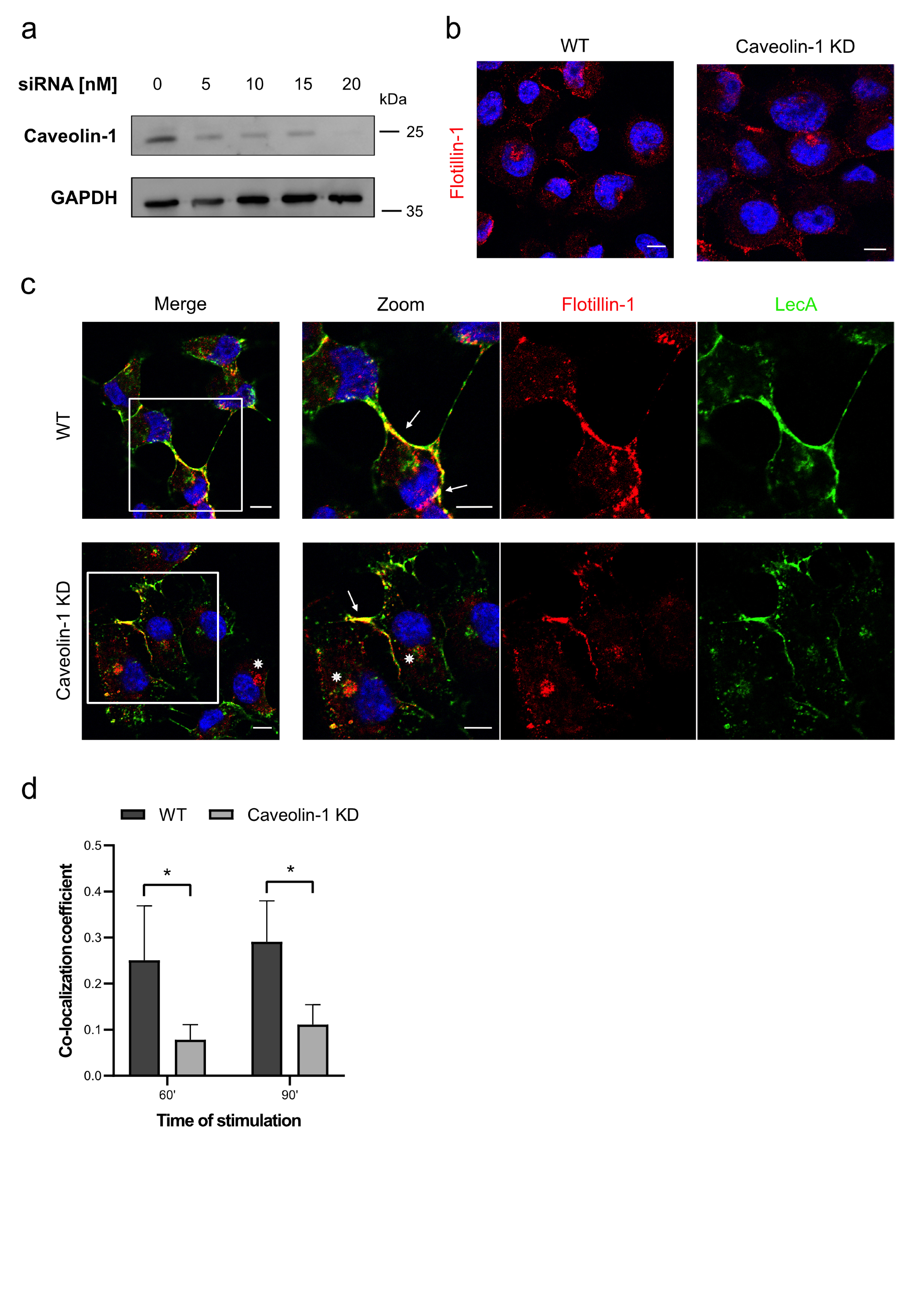


**Fig. S7** Re-localization of flotillin-1 to the plasma membrane upon LecA stimulation is attenuated in caveolin-1-depleted cells. **a** Caveolin-1 is knocked-down by siRNA treatment in a dose-dependent manner. Subsequent experiments were performed in H1299 cells transfected with caveolin-1 siRNA at a concentration of 20 nM. **b** Flotillin-1 staining in unstimulated H1299 WT and caveolin-depleted cells. Nuclei were counterstained by DAPI. Scale bar: 10 μm. **c** Fluorescence co-localization studies of flotillin-1 (red) and LecA (green) after 90 min of LecA stimulation. Nuclei were counterstained by DAPI. Framed areas were magnified. White arrows point at co-localization events at the plasma membrane, asterisks at perinuclear localization ­of flotillin-1. Scale bar: 10 μm. **d** Co-localization between LecA and flotillin-1 in WT and caveolin-1-depleted cells was quantified by Mander’s co-localization coefficient. Bars display mean values of at least three biological replicates, error bars represent SD, *p<0.05 (one-way ANOVA and Sidak’s multiple comparisons test). KD: Knock-down.

**Fig. S8** PI3-kinase inhibition partially prevents PIP_3_ clustering and recruitment of flotillins upon LecA stimulation. **a** Confocal microscopy images of PH-Akt-GFP and flotillin-2-mCherry expressing H1299 cells exposed to fluorescent LecA. Lower two panels: Cells were pre-treated with 100 nM Wortmannin to inhibit PI3-kinase activity. Scale bar: 10 μm. **b** Co-localization of PH-Akt-GFP and LecA is depicted as Mander’s co-localization coefficient normalized to the untreated conditions. **c** Mander’s co-localization coefficient quantified between the fluorescent signals of flotillin-2-mCherry and LecA in comparison to the untreated conditions. Bars display mean values of three biological replicates, error bars represent SD, **p<0.01, ***p<0.001, ****p<0.0001 (two-way ANOVA and Tukey’s multiple comparisons tests).

### **Fig. S9** CRISPR-Cas9 knockout of flotillin-1. Flotillin-1 knockout efficiency of two clones was analyzed by qPCR and comparison of mRNA expression levels. For further experiments, clone 52 was chosen, as flotillin-2 mRNA expression appeared similar to WT levels. Bars display mean values of two biological replicates, error bars represent SD.

### **Table S1** Sequences of used siRNAs, primers for qPCR and sgRNAs for CRISPR-Cas9 knockout experiments.

### **Table S2** The MS protein hits: The MS protein analysis revealed a list of hits mainly including cytoskeleton and cytoskeletal-related components as well as small GTPases. Hits are ordered according to abundance. Listed are only proteins detected in both replicates and present with an emPAI >0.01%.
