# Supplementary material for: The Gb3-enriched CD59/flotillin plasma membrane domain regulates host cell invasion by Pseudomonas aeruginosa
Source: Cell Mol Life Sci. 2021 Feb 8;78(7):3637–56. doi: 10.1007/s00018-021-03766-1 (PMC8038999; doi:10.1007/s00018-021-03766-1)
Supplement: Supplementary file 14 — Supplementary file14 (DOCX 12 KB) [file 18_2021_3766_MOESM14_ESM.docx]

**Online Resource 1** PH-Akt-GFP (as a sensor for PIP3) and LecA co-localize in live cell-microscopy experiments of stimulated H1299 cells. PH-Akt-GFP expressing H1299 cells were exposed to fluorescent LecA and PIP3 clustering was followed live. White arrows point at dynamic endocytic events of LecA that strongly co-localize with PH-Akt-GFP. Scale bar: 10 μm. Time of stimulation is indicated in minutes.
